# Real-time quantification of protein expression at the single-cell level via dynamic protein synthesis translocation reporters

**DOI:** 10.1038/ncomms11304

**Published:** 2016-04-21

**Authors:** Delphine Aymoz, Victoria Wosika, Eric Durandau, Serge Pelet

**Affiliations:** 1 Department of Fundamental Microbiology, University of Lausanne, Lausanne CH-1015, Switzerland

## Abstract

Protein expression is a dynamic process, which can be rapidly induced by extracellular signals. It is widely appreciated that single cells can display large variations in the level of gene induction. However, the variability in the dynamics of this process in individual cells is difficult to quantify using standard fluorescent protein (FP) expression assays, due to the slow maturation of their fluorophore. Here we have developed expression reporters that accurately measure both the levels and dynamics of protein synthesis in live single cells with a temporal resolution under a minute. Our system relies on the quantification of the translocation of a constitutively expressed FP into the nucleus. As a proof of concept, we used these reporters to measure the transient protein synthesis arising from two promoters responding to the yeast hyper osmolarity glycerol mitogen-activated protein kinase pathway (p*STL1* and p*GPD1*). They display distinct expression dynamics giving rise to strikingly different instantaneous expression noise.

## Abstract (web summary)

Single cells can display large heterogeneity in gene induction. Here, Aymoz *et al*. present an expression reporter based on protein translocation that can accurately measure both the levels and dynamics of protein synthesis in live single cells with a temporal resolution of less than one minute.

## Graphical abstract

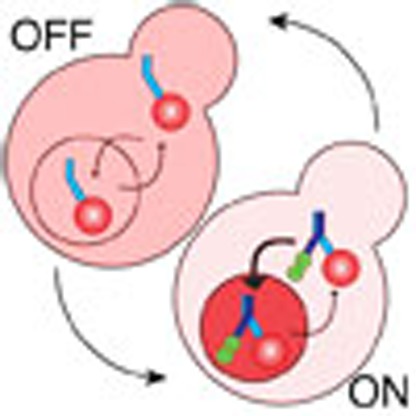

Protein synthesis is a multi-step process. It is typically initiated by the activation of a transcription factor (TF), which binds the promoter sequence of a gene. This active TF allows the recruitment of the polymerase resulting in the formation of an initiation complex. In parallel, chromatin-remodelling enzymes act on the locus to enable the efficient transcription of the gene. The polymerase travels along the locus to produce the mRNA. After transcription, the mRNA is exported out of the nucleus to be translated into the amino acid chain that will form the protein. Many complexes and enzymes implicated in this process have been characterized, allowing a detailed mechanistic understanding of the entire protein expression machinery^1^,^2^. Comparatively, little is known about how a given DNA sequence influences the final amount of protein produced, the dynamics at which it is expressed or the cell-to-cell variability in the level of protein synthesized. Since the promoter sequence of a gene controls the first steps in protein synthesis, it plays a key role in controlling the final protein levels^1^,^2^,^3^,^4^,^5^,^6^.

To quantify mRNA and protein levels, numerous techniques have been developed. However, most of these measurements are performed at the population level (northern blots, western blots) and/or provide snapshots of the cell content at a given point in time (flow cytometry). Only live-cell microscopy combined with fluorescent protein (FP)-based technologies provides a tool to quantify, at the single-cell level, the temporal evolution of protein expression in a given cell.

Using this technique, Elowitz *et al*.^7^ measured for the first time the noise associated with protein production using a set of two FPs controlled by identical promoters. To dissect the origin of the fluctuations in protein levels in the same cell, they defined the intrinsic and extrinsic expression noises. This allowed them to observe that the variability between individual cells (extrinsic) as well as stochastic intracellular processes (intrinsic) contribute to the total expression noise.

Many studies have since used FP variants to quantify expression levels in individual cells, either by fusing the FP to a target protein or by placing the FP under the control of a promoter of interest^8^,^9^,^10^,^11^. Unfortunately, the maturation time of FP, which varies from tens of minutes to more than an hour, sets a low limit to the dynamics that can be observed^12^,^13^. In many signalling pathways, the appropriate timing of gene expression is tightly controlled since it can influence the output of the system^14^,^15^,^16^. Moreover, the stable fold of the FP results in a very long half-life in the cell, which hinders the monitoring of oscillatory or transient protein synthesis.

To circumvent these problems, we set out to design a new assay to monitor protein expression induced by a promoter element based on the relocation of a constitutively expressed FP, thereby avoiding the maturation delay. We report here on the development and validation of the dynamic protein synthesis translocation reporter (dPSTR), that provides real-time measurement of protein expression arising from a promoter element in live single cells. As a proof of concept, we adapted the dPSTR system for the study of the protein production driven by the hyper osmolarity glycerol (HOG) pathway in the model organism *Saccharomyces cerevisiae*. We show that by measuring the relocation of an FP into the nucleus, we are able to accurately and dynamically quantify protein expression in hundreds of single cells, with a time resolution under a minute. We also demonstrate that two dPSTRs can be combined in the same strain allowing the first real-time measurements of intrinsic and extrinsic expression noises. Finally, we further prove the dynamic nature of this assay by stimulating cells multiple times to quantify successive rounds of protein expression.

## Results

### Design of the dynamics expression reporter

**Figure 1:**
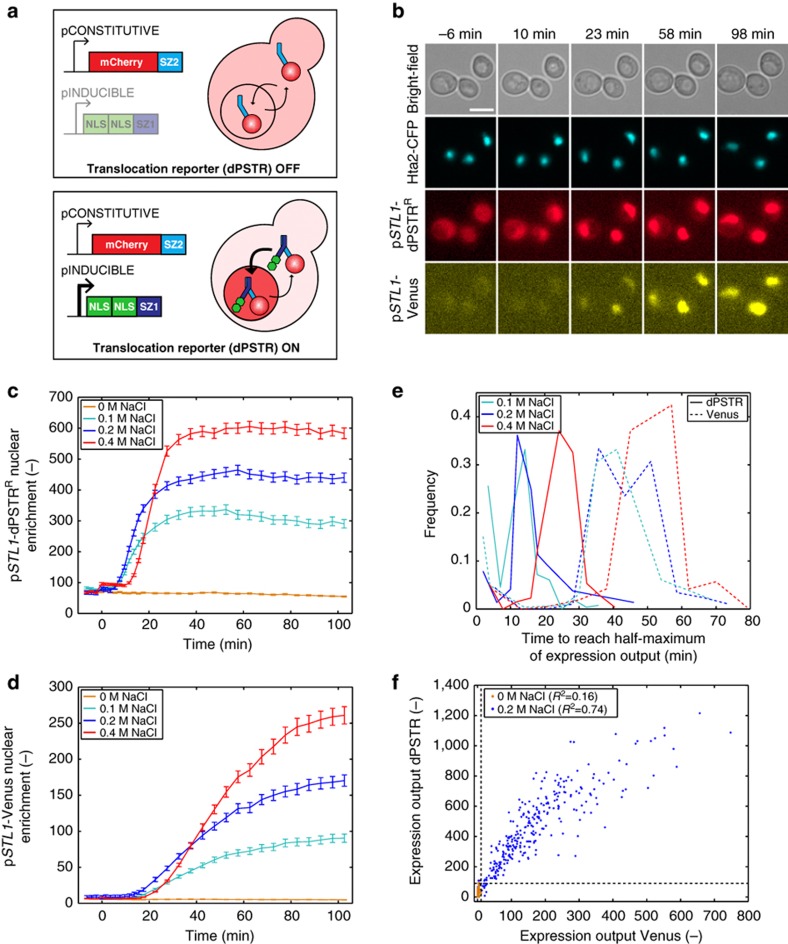
Dynamic measurements of protein synthesis with a translocation reporter. (**a**) A FP is fused to a SynZip (SZ2), expressed under the control of a constitutive promoter and can freely diffuse between the cytoplasm and the nucleus (dPSTR OFF, top). The induction of the promoter of interest drives the expression of the second peptide of the reporter, composed of two NLSs fused to a compatible SynZip (SZ1). The strong interaction between the SynZip peptides leads to the enrichment of the FP in the nucleus (dPSTR ON, bottom). (**b**) Microscopy images of cells with histone Hta2 tagged with CFP and carrying the p*STL1*-dPSTR^R^ submitted to a 0.2 M NaCl stress. The inducible peptide is fused to a Venus FP. Scale bar, 5 μm. (**c**,**d**) Quantifications of the nuclear enrichment in the dPSTR^R^ (**c**) and the Venus (**d**) channels for cells stressed with 0 (orange, *N*_C_=285), 0.1 (cyan, *N*_C_=266), 0.2 (blue, *N*_C_=294) or 0.4 M NaCl (red, *N*_C_=265). Nuclear enrichment is measured as the difference between nuclear and cytoplasmic fluorescence for each single cell. For all similar graphs throughout the paper, the solid lines represent the population average and the error bars are the s.e.m. *N*_C_ represents the number of single cells measured. (**e**) Histograms of the time needed for each single cell to reach half of its expression output for either the dPSTR^R^ (solid lines) or the Venus (dashed lines). The expression output represents the maximal amplitude of the nuclear enrichment (see Methods). (**f**) Single cell correlation of the expression output measured by either the p*STL1*-dPSTR^R^ or the p*STL1*-Venus assay, for control cells (orange) or cells induced with 0.2 M NaCl (blue). The dashed lines represent the expression thresholds, above which cells are considered as expressing. All the figures of the paper represent one representative experiment of at least three biological replicates.

As the maturation time of an FP hinders the quantification of the dynamics of protein expression, we designed an expression reporter that bypasses this rate-limiting step. Our assay uses the change in subcellular localization of a mature FP as a read-out of protein synthesis driven by a promoter of interest. The dPSTR is a protein heterodimer which is encoded by two transcriptional units present on a unique plasmid integrated in the yeast genomic DNA. The first unit constitutively expresses the FP that can freely diffuse between the nucleus and the cytoplasm. The second unit is under the control of the promoter of interest and encodes two nuclear localization signals (NLS), which promote the active import of proteins into the nucleus^17^. The interaction between the FP and the NLSs is driven by the presence of synthetic bZip domains (SynZips or SZ) in each unit^18^,^19^. These SynZips form strong and specific heterodimers that induce a relocation of the FP into the nucleus, proportionally to the expression level of the NLSs (Fig. 1a).

### Validation of the method

As a proof of concept, we integrated a dPSTR measuring the expression of the osmostress responsive promoter p*STL1* by the relocation of the red fluorescent protein (RFP) variant mCherry (p*STL1*-dPSTR^R^, ^R^ denotes the fluorescent channel used: RFP), in a strain bearing a histone tagged with cyan fluorescent protein (CFP). Hyper-osmotic shock triggers the transient activation of the HOG pathway, which culminates in the activation of the mitogen-activated protein kinase (MAPK) Hog1. When activated, Hog1 increases the intracellular glycerol production, driving the adaptation of the cells to the high-osmolarity medium. In addition, Hog1 induces the transcription of 300–700 stress response genes^20^,^21^,^22^. Among them, *STL1* has been used as a model for stress activated gene expression and is widely studied^11^,^23^,^24^,^25^.

To demonstrate the validity of our approach and compare it to traditional expression reporters, we fused a fast maturing Venus FP^26^ to the inducible construct controlled by p*STL1* (2xNLS-Venus-SZ). Before induction, the RFP signal is homogeneously distributed between the nucleus and the cytoplasm and no Venus fluorescence can be detected (Fig. 1b). Upon addition of NaCl, the HOG pathway is activated and the inducible part of the dPSTR is expressed. This results in a detectable enrichment of the mCherry in the cell nucleus 10 min after induction, while at the same time point, no fluorescent signal from the Venus can be detected. Note that this nuclear enrichment is dependent on the formation of the SZ heterodimer and on the nature of the promoter element (Supplementary Fig. 1a,b).

To quantify the dynamics of protein production, time-lapse movies for three different concentrations of salt (0.1, 0.2 and 0.4 M NaCl) and a control were measured in parallel. Using an automated image analysis pipeline^27^, the nucleus and cytoplasm of the cells were segmented and tracked during the entire experiment. At each time point, their average fluorescence intensity was measured (Supplementary Fig. 2). Figure 1c represents the temporal evolution of the difference between nuclear and cytoplasmic fluorescence in RFP, which is a measure of the level of dPSTR nuclear enrichment. Note that the small increase in nuclear enrichment happening at time zero is an artefact of the shrinking of the cells upon hyper-osmotic stress addition and is not a transcriptional response of the cell (Supplementary Fig. 1a,b). The dynamics in dPSTR nuclear enrichment can be compared with those obtained for the expression of the Venus FP (Fig. 1d). Although the graded protein production due to increasing salt concentration is observed with the two methods, there is a clear kinetic difference between the dPSTR behaviour and the Venus fluorescence signal. The latter appears with a delay, rises more slowly, and reaches its maximum later. We attribute this difference to the maturation step required to form the FP fluorophore^26^,^28^.

To quantify this difference more precisely, we measured the time when half of the maximal nuclear enrichment of each FP was obtained for each single-cell trace (Fig. 1e, see Methods). At 0.2 M NaCl, with the dPSTR sensor, the majority of cells need between 10 and 20 min to reach this value, while the half-maximum of the Venus fluorescence signal is reached later and with a larger spread (between 30 and 60 min). The delay in protein production at 0.4 M NaCl relative to the other concentrations has been attributed to a strong compression of the cell^29^. This temporal difference is clearly identified with the dPSTR, while with Venus expression, the distributions at 0.2 and 0.4 M are overlapping, indicating similar kinetics of expression for many cells.

To verify whether the dynamics measured with the dPSTR reflected the real kinetics of protein production, we used flow cytometry, a method that provides snapshot measurements of the dynamics of protein expression^30^. The cells bearing the expression reporter p*STL1*-qVenus were treated with NaCl. At specific time points, translation was blocked by addition of cycloheximide. All the qVenus produced at that point was allowed to mature for 2 h before the measurement was performed. The evolution of protein production quantified by this method aligns well with the live-cell measurements performed with the dPSTR (Supplementary Fig. 1c), showing that the dPSTR quantifies the expression dynamics precisely. Indeed, both dPSTR and flow cytometry measurements indicate that proteins start to be synthesized 10 min after the hyper-osmotic stress.

While the dPSTR provides a faster and more accurate determination of the expression kinetics, we wanted to verify whether the level of protein expression measured with the dPSTR was comparable to the one measured with the classical promoter-FP fusion. By setting a threshold based on the non-induced control, we verified that the percentage of expressing cells in the population based on the p*STL1*-dPSTR^R^ or p*STL1*-Venus signals provided similar proportions (Supplementary Fig. 3a,b). Figure 1f also demonstrates that there is a high correlation (*R*^2^=0.74 at 0.2 M) between the amounts of Venus measured and the nuclear enrichment of the dPSTR. Note that this correlation is higher at 0.1 M and slightly drops at 0.4 M (Supplementary Fig. 3c–e). This difference could be explained by a saturation effect of the nuclear enrichment of the sensor (Supplementary Note 1 and Supplementary Fig. 4). Taken together, these results demonstrate that the relocation of the FP in the dPSTR assay provides a real-time measurement of protein synthesis in live single cells, allowing accurate quantification of both levels and kinetics of protein expression arising from a promoter of interest.

### Transient expression

**Figure 2:**
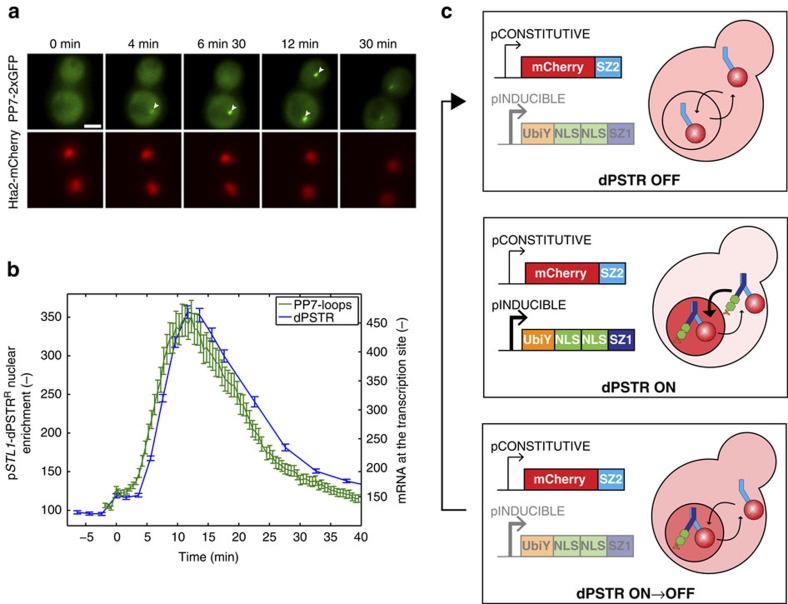
Measurements of transient gene expression. (**a**) Maximum intensity projections from Z-stacks of cells bearing an Hta2-mCherry tag and transformed with the PP7-2xGFP system with 24 mRNA PP7-stem loops under the control of the *STL1* promoter stimulated with 0.2 M NaCl. The presence of transcription site in the nucleus is highlighted by white arrowheads. Scale bar represents 5 μm. (**b**) Comparison between the mRNA apparition at the transcription site using the PP7-2xGFP (green, *N*_C_=285) and the unstable p*STL1*-dPSTR^R^ nuclear enrichment (blue, *N*_C_=655) in two different strains, under stimulation by 0.2 M NaCl. The fluorescence of the transcription site was quantified by measuring the difference between the 20 brightest pixels in the nucleus and the average nuclear fluorescence. (**c**) The dPSTR was modified to measure transient gene expression by addition of an UbiY destabilization sequence at the N-terminus of the induced peptide (2xNLS-SZ). The degradation of the induced construct allows the FP to recover its initial homogenous distribution throughout the cell after stimulation.

Environmental stresses cause a profound but transient modification in the yeast transcriptional program^21^. Northern blot measurements confirmed that the *STL1* mRNAs are produced within 4 min after hyper-osmotic shock but remain in the cell for less than an hour, in agreement with the transient activation of the HOG pathway^20^,^24^. To obtain a more precise estimate of the dynamics of mRNA production arising from p*STL1*, we implemented the PP7 system, which allows the identification of the transcription site as a bright fluorescent focus in the nucleus^31^ (Fig. 2a). Twenty-four mRNA stem loops, placed under the control of the *STL1* promoter, are recognized by the bacteriophage coat protein PP7 tagged with a double-GFP. Upon induction of the cells with NaCl, bright fluorescent dots appears in a majority of the nuclei. Quantification of the intensity of these nuclear foci provides a dynamic read-out of the mRNA production (Fig. 2b). Upon 0.2 M NaCl stress, foci can be observed in few cells already 3 min after stress. The intensity and the number of the nuclear foci tend to decrease 10 min after induction. The delay between the stimulus and mRNA production corresponds to the time required for signal transduction, association of Hog1 with TFs, induction of chromatin remodelling and recruitment of the polymerase^24^,^32^.

The PP7 measurements confirm the transient nature of the transcription induced by the activation of the HOG pathway. To obtain a read-out of protein synthesis induction and arrest, we modified the inducible unit by adding a destabilization sequence (UbiY-2xNLS-SZ, Fig. 2c). Upon translation of the peptide, the leading ubiquitin is cleaved off and the exposed amino acid (Y) decreases the half-life of the protein to a few minutes^33^. With this construct, protein production is counterbalanced by protein degradation. As long as the rate of protein production is larger than protein degradation, the dPSTR accumulates in the nucleus (Fig. 2b). Shortly after the mRNA production reaches its maximum, the dPSTR fluorescence which has accumulated in the nucleus starts to return slowly to a uniform localization as can be seen by the decline in nuclear enrichment. Moreover, using the unstable version of the dPSTR prevents the accumulation of the inducible peptide in the cell thereby avoiding any saturation effect (Supplementary Figure 5a–c).

Using cycloheximide inhibition, we quantified a half-life for the unstable peptide of 2 min (Supplementary Fig. 5d). Therefore, the observed decline in nuclear enrichment, with a half-life close to 10 min, is not only solely limited by the dPSTR degradation rate but also reflects the implication of other biological factors, such as the arrest of transcription and the stability of the mRNA. The comparison between the PP7 and dPSTR measurements shows a short expected temporal delay between mRNA transcription and protein synthesis comprising processes such as mRNA export and translation^34^. This close consecutive apparition of PP7 and dPSTR signals further confirms that the dynamics of protein production measured with the dPSTR correspond to the genuine kinetics of protein expression.

### Correlation of signalling activity and protein expression

**Figure 3:**
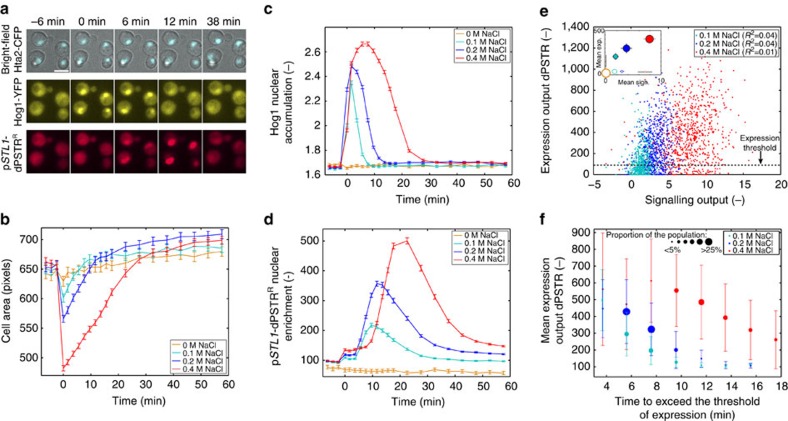
Lack of correlation between Hog1 activity and p*STL1* expression at the single-cell level. (**a**) Microscopy images of a strain with Hog1 tagged with mCitrine and carrying the unstable p*STL1*-dPSTR^R^ that was challenged by 0.2 M NaCl. The nuclear accumulation of Hog1-YFP precedes protein expression. Scale bar, 5 μm. (**b**–**d**) Quantification of the cell area (**b**) Hog1 nuclear accumulation, measured as the ratio between nuclear and cytoplasmic YFP fluorescence (**c**) and p*STL1*-dPSTR^R^ nuclear enrichment (**d**) for cells stimulated with 0 (orange, *N*_C_=467), 0.1 (cyan, *N*_C_=558), 0.2 (blue, *N*_C_=655) and 0.4 M NaCl (red, *N*_C_=802). (**e**) Scatter plot of the signalling output measured as the integral below the Hog1 nuclear accumulation curve versus the expression output measured as the maximum in p*STL1*-dPSTR^R^ nuclear enrichment. The dashed line represents the expression threshold. The mean signalling output versus the mean expression output for the expressing cells (filled circles) and the non-expressing cells (empty circles) is plotted in the inset. The size of the marker is indicative of the percentage of cell in each category. (**f**) Correlation between the time needed to overcome the expression threshold and the expression output. The mean expression output and the standard deviation were calculated for groups of cells, which exceed the expression threshold at the same time point. The marker size is indicative of the percentage of cells (from the total population) in each group.

In order to correlate signalling activity and protein expression dynamics, the degradable reporter construct (comprising the UbiY destabilization sequence) was transformed in a strain bearing the MAPK Hog1 tagged with yellow fluorescent protein (YFP) (Fig. 3a). Hog1 nuclear accumulation upon hyper-osmotic stress is linked to its activity^35^ and has been extensively used to quantify the dynamics of signal transduction in the HOG pathway^11^,^36^,^37^. A few minutes after Hog1 relocates in the nucleus, the p*STL1*-dPSTR^R^ starts to accumulate in the nucleus.

Figure 3b displays the changes in cell area upon increasing osmotic challenges, which trigger an immediate shrinking of the cells. Depending on the severity of the stress, the cells need between 10 and 30 min to recover their original sizes. Figure 3c depicts Hog1 relocation, quantified as the ratio of nuclear to cytoplasmic YFP fluorescence as a function of time, which is almost a mirror image of the cellular adaptation process. Indeed, Hog1 enters the nucleus quickly after stress, and it returns to a uniform localization when cells recover their original sizes. The MAPK drives the adaptation process by increasing the production of glycerol, causing a negative feedback on its own activity^38^,^39^.

In comparison, the dynamics of protein production measured by the p*STL1*-dPSTR^R^ is delayed because a number of events need to be completed before proteins can be produced (Fig. 3d). These biological processes include promoter activation, which can require TF recruitment and chromatin remodelling, and mRNA synthesis and translation^40^. The maximal protein production, corresponding to the peak in nuclear enrichment, is reached when Hog1 returns to its basal level. It has been shown that active Hog1 is closely associated with all the steps of transcription and even travels along the ORFs with the elongation complex^40^. Therefore, transcription is expected to stop as soon as Hog1 activity returns to its basal level.

When measured at the population level, both Hog1 activity and p*STL1* expression increase with the strength of the stress; however, this correlation does not hold true for single-cell measurements (Fig. 3e). This discrepancy has been attributed to slow chromatin-remodelling steps occurring at the promoter, which decouple the expression from the level of MAPK activity^11^. One prediction from such a model is that cells that become transcriptionally active earlier tend to express more proteins, since they profit from a longer temporal window of gene expression for the same MAPK activity. Indeed, it can be seen in Fig. 3f that there is an inverse correlation between the time when the protein production is detected and the level of protein produced. The unique ability of the dPSTR to measure the dynamics of protein production in real time allows us to confirm that there is a direct influence of the time when the promoter is activated on the output in protein production.

Similar experiments were performed with another stress-inducible promoter p*GPD1* (Supplementary Fig. 6; refs 41, 42). In comparison with p*STL1*, which is repressed under normal growth conditions, p*GPD1* has a low basal level of transcription, which is increased under conditions of hyper-osmotic stress in a Hog1-dependent manner. The combined measurements of the Hog1-YFP relocation dynamics and the p*GPD1*-dPSTR^R^ expression provided in general a very similar picture to the p*STL1* measurements. One obvious difference is that a larger fraction of the population expresses the p*GPD1*-dPSTR^R^ at low stress levels (Inset in Supplementary Fig. 6e). In addition, we noticed that p*GPD1*-dPSTR^R^ seems to be expressed more rapidly than the p*STL1*-dPSTR^R^ (compare panels d and f from Supplementary Fig. 6 to those of Fig. 3).

**Figure 4:**
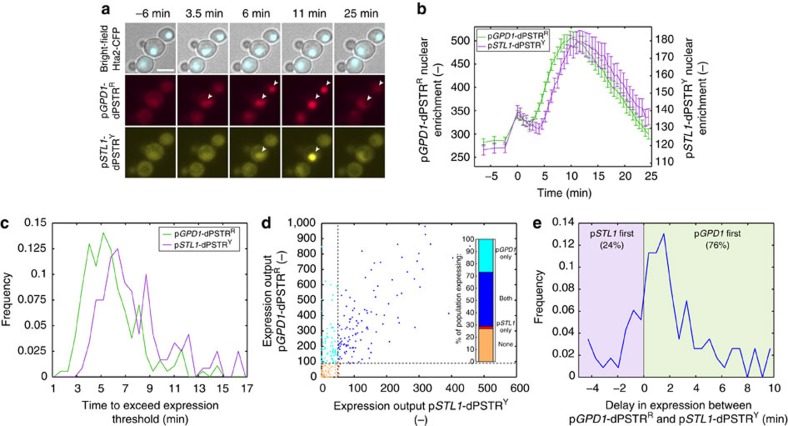
Dynamic measurements of the induction of two osmostress responsive promoters in the same cells. (**a**) Microscopy images of cells carrying p*GPD1*-dPSTR^R^ (RFP channel) and p*STL1*-dPSTR^Y^ (YFP channel) stimulated with 0.2 M NaCl. The two reporters are built with two orthogonal pairs of SynZips. The arrowheads indicate the nuclei with accumulated FPs, highlighting the different timings of accumulation of each dPSTR. Scale bar, 5 μm. (**b**) Quantification of the nuclear enrichment of p*GPD1*-dPSTR^R^ (green, left axis) and p*STL1*-dPSTR^Y^ (purple, right axis) in course of time (*N*_C_=260). (**c**) Histograms of the time needed to overcome the expression threshold for cells expressing the indicated promoter. (**d**) Correlation between the expression output of p*GPD1*-dPSTR^R^ and the one of p*STL1*-dPSTR^Y^ in single cells (*R*^2^=0.48). The dashed lines represent the expression thresholds for each dPSTR. The inset is showing the fraction of the population expressing either p*GPD1* alone (cyan), p*STL1* alone (red), both promoters (blue) or none (orange). (**e**) The delay between p*GPD1* and p*STL1* expression in cells that express both dPSTRs calculated from the difference in time to overcome the expression threshold for both reporters. Positive times represent cells where p*GPD1* overcomes the expression threshold first (green area, 76% of the cells expressing both promoters), and negative or null times indicates that p*STL1* is expressed before or at the same time as p*GPD1* (purple, 24%).

To better characterize this difference in expression dynamics between the two stress-inducible promoters, we combined a p*GPD1*-dPSTR and a p*STL1*-dPSTR in the same cells (Fig. 4a). This combination is possible because they possess two sets of orthogonal SynZips (SZ1/SZ2 and SZ3/SZ4 (ref. 19)) and drive the relocation of either a red or a yellow FP variant (resp. p*GPD1*-dPSTR^R^ and p*STL1*-dPSTR^Y^). Following a 0.2 M stimulus, the cells were imaged with 35 s resolution (Fig. 4b). The average response of the population indicates a 1.5 min delay between p*GPD1* and p*STL1* expression in favour of p*GPD1* (Fig. 4c). This delay remains constant during the complete period of expression of the two promoters. Moreover, since both reporters are present in the same cell, we can correlate their expression output within individual cells. Approximately a third of the cells expressing p*GPD1* do not express p*STL1*, while only a few cells were identified as p*STL1* positive only (Fig. 4d). In the cells expressing both reporters, we could observe that p*GPD1* expression precedes p*STL1* expression in a large majority of cells (Fig. 4e). We can infer that this temporal delay observed for two promoters within the same cell, which are thereby experiencing the same level of Hog1 activity and are controlled by the same TF Hot1 (ref. 22), can be attributed either to different efficiency of the TFs associated with each promoters to recruit the transcriptional machinery or to the chromatin remodelling step. We propose that the important remodelling taking place on the repressed p*STL1* promoter^24^ is largely absent from the p*GPD1*.

### Dynamic noise quantification

**Figure 5:**
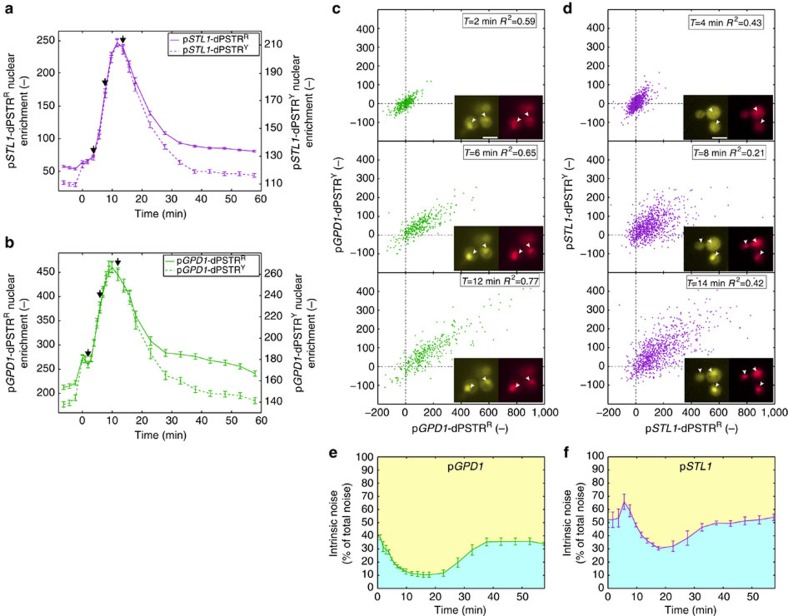
Measurement of the dynamic evolution of intrinsic and extrinsic expression noise of p*STL1* and p*GPD1*. (**a**,**b**) Quantification of the nuclear enrichment of dPSTR^R^ (right axis) and dPSTR^Y^ (left axis) for a strain carrying two p*STL1*-dSPTRs (**a**) or two p*GPD1*-dPSTRs (**b**) in cells stimulated with 0.2 M NaCl (resp. *N*_C_=958 and *N*_C_=368). (**c**,**d**) Correlation of the instantaneous corrected nuclear enrichment of p*GPD1*-dPSTRs (**c**) or p*STL1*-dPSTRs (**d**) in YFP and RFP channels at the indicated times after induction. Pictures indicate representative cells at the same time points. Arrowheads are highlighting nuclei in the focal plane Scale bars, 5μm. (**e**,**f**) Evolution of the intrinsic expression noise for p*GPD1* (**e**) and p*STL1* (**f**). The blue area under the curve represents the proportion of intrinsic noise, and the yellow area above represents the proportion of extrinsic noise.

A prediction from our observations and from previous studies^11^,^25^ is that slow stochastic chromatin remodelling is responsible for a large intrinsic noise in p*STL1* expression. Indeed, the recruitment of the chromatin remodelling machinery is thought to occur stochastically at each locus, creating large variability in expression within the same cell. However, this variability should be mostly absent from the p*GPD1*-dependent expression. To monitor the temporal fluctuations of this noise, we combined two dPSTRs in the same cell controlled either by two *STL1* promoters or two *GPD1* promoters (Fig. 5a,b and Supplementary Fig. 7). The absolute intensity of nuclear relocation measured in both channels is different, due to disparities in FP brightness, but the dynamics of relocation are similar for both reporters (Supplementary Fig. 8). For each time point in the data set, we can correlate the instantaneous amplitude of nuclear relocation in the yellow and in the red channels in every single cell. Three time points in the early, intermediate and highest phase of expression have been selected (arrows in Fig. 5a,b) and are plotted in Fig. 5c,d. It is apparent that throughout the time-lapse, the two p*GPD1*-dPSTRs display a very tight correlation, which is largely absent from the double-p*STL1*-dPSTRs strain. For example, the upper cell shown in panel d is expressing first the p*STL1*-dPSTR^R^ copy, and only later the p*STL1*-dPSTR^Y^. Thus, both the dynamics and the levels of expression can significantly vary between the two p*STL1*-dPSTRs within the same cell.

Using these data, we calculated the evolution of the intrinsic expression noise over time^7^,^8^ (Fig. 5e,f; see Methods). In the p*GPD1* case, at time zero, the intrinsic noise is relatively low and drops further as the two dPSTRs are expressed synchronously. Interestingly, in the p*STL1* case, protein production can arise stochastically from either locus, resulting in an initial increase in the proportion of intrinsic noise. Later on, as the two loci are expressed, this component of the noise tends to decrease, because transcripts in the same cell share the same translational machinery. The expression capacity of a cell, which is linked to the number of ribosomes, is thought to be a major determinant of extrinsic noise^10^. A similar behaviour can be observed with cells induced with 0.1 and 0.4 M NaCl (Supplementary Fig. 9). To conclude, the dPSTR system allows the accurate measurement of the evolution of the expression noise in real time, which cannot be accomplished using other current methods.

### Induction of successive rounds of protein expression

**Figure 6:**
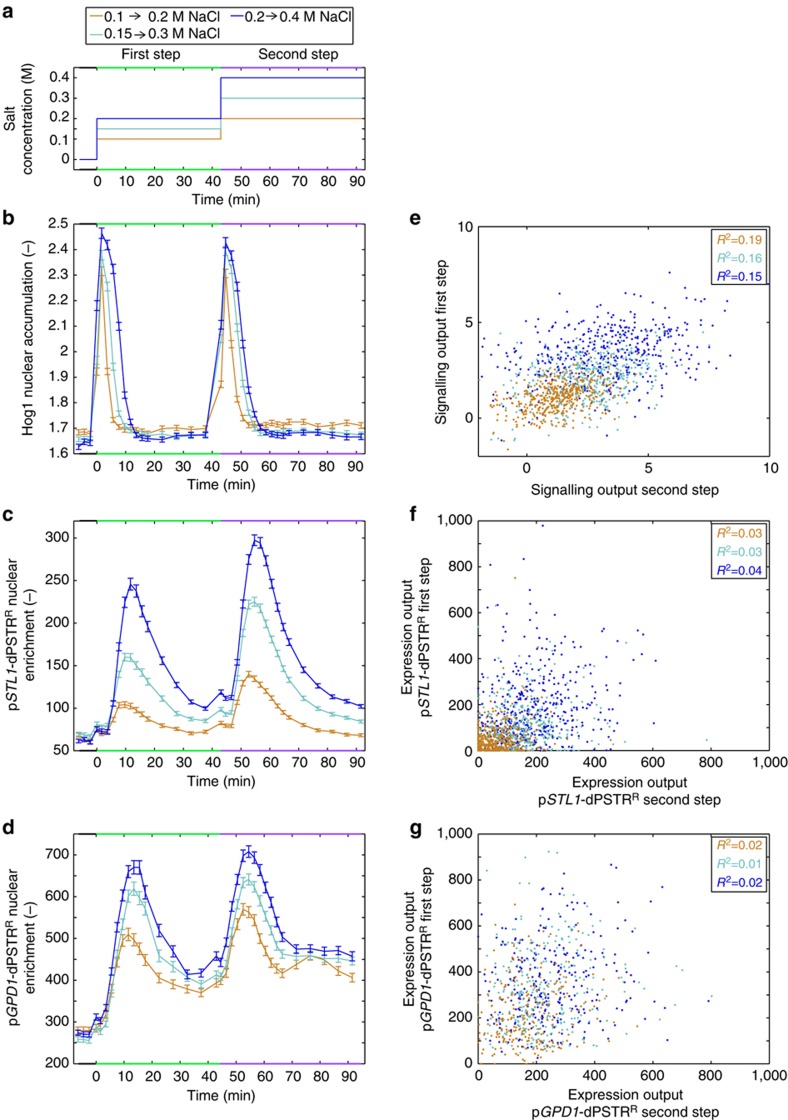
Consecutive hyper-osmotic stresses result in uncorrelated transcription events. (**a**) Evolution of the NaCl concentration over time. Cells were stimulated at time 0 with a given concentration of NaCl. A second hyper-osmotic stress of similar amplitude was performed 43 min later by doubling the concentration of NaCl in the well: 0.1→0.2 (orange), 0.15→0.3 (cyan), 0.2→0.4 (blue) (throughout the entire figure). (**b**,**c**) Quantification of the average Hog1 nuclear accumulation (**b**) and p*STL1*-dPSTR^R^ nuclear enrichment (**c**) for cells subjected to the steps in NaCl concentrations depicted in (**a**) (0.1→0.2: *N*_C_=429; 0.15→0.3: *N*_C_=450; 0.2→0.4: *N*_C_=449). (**d**) Quantification of the average p*GPD1*-dPSTR^R^ nuclear enrichment, from a different strain, subjected to the steps in NaCl concentrations depicted in **a** (0.1→0.2: *N*_C_=235; 0.15→0.3: *N*_C_=296; 0.2→0.4: *N*_C_=276). These cells also bear Hog1-YFP that showed the same behaviour as in **b**. (**e**–**g**) Correlations of the signalling outputs (**e**), of the expression outputs of p*STL1*-dPSTR^R^ (**f**) or of the expression outputs of p*GPD1*-dPSTR^R^ (**g**) for the two stress events.

The property of the dPSTR to return to its initial cytoplasmic distribution after degradation of the induced moiety should allow the measurement of multiple rounds of protein expressions. To test this, cells bearing a Hog1-YFP combined either with a p*STL1*-dPSTR^R^ or a p*GPD1*-dPSTR^R^ were subjected to a first hyper-osmotic stress. Forty-three minutes later, the NaCl concentration was further increased to double the osmolarity in the medium (Fig. 6a). The two hyper-osmotic events led to two shrinking and recovery phases driven by the activation of Hog1 (Fig. 6b and Supplementary Fig. 10a). Each period of Hog1 activity resulted in p*STL1*-dPSTR^R^ and p*GPD1*-dPSTR^R^ relocation (Fig. 6c,d). At the population level, there is a linear correlation between Hog1 signalling output and the expression output of the two promoters, which is maintained for the first step and the second step of stimulation (Supplementary Fig. 10b,c).

Interestingly, the level of Hog1 activity in individual cells is weakly correlated between each stress (Fig. 6e). Cells that have responded strongly in the first step are also more likely to respond strongly in the second step. As expected from our previous single induction experiments, no single cell correlation between Hog1 activity and subsequent protein expression is observed in either pulse, neither for p*STL1* nor for p*GPD1* (Supplementary Fig. 10d–g). Because of the stochastic activation induced by the chromatin-remodelling step, the p*STL1* expression cannot be correlated between each pulse (Fig. 6f). We can identify cells that responded to the first pulse but not the second one or vice-versa, or cells that responded to both steps or not at all. This large diversity in responses demonstrates that the ORF does not retain a memory of previous transcription events. This is in agreement with previous studies which have demonstrated that histones are reassembled rapidly once the transcription has stopped in order to repress the locus^43^. It is however more surprising to see that the p*GPD1* expression in the first and second pulse is not correlated either (Fig. 6g). A large majority of the cells express in both pulses but do it to a different extent. This suggests that the cellular parameters that allowed a strong correlation of the *GPD1* expression during a single pulse (Fig. 5) are not maintained from one stimulus to the next to allow a correlation across time (Fig. 6g).

## Discussion

In this paper, we have demonstrated the use of a synthetic translocation reporter to quantify the dynamics of protein synthesis emanating from a promoter of interest in live single cells. The clear advantage of the dPSTR system is that it provides measurements at the minute timescale of protein expression events that FP cannot offer, due to the slow maturation time of the fluorophore. Even the fast folding sfGFP needs on average 6 min to become fluorescent^44^,^45^, which precludes the fast measurement of the protein expression dynamics. Moreover, there is no comparable fast maturing FP in other spectral channels, limiting this technique to only one promoter in a given strain. In our assay however, the quantification of the dynamics of protein expression are limited only by the import rate into the nucleus, which happens on the sub-minute timescale^46^. Since the assay relies on constitutively expressed FPs, the FP can be easily exchanged without affecting the dynamics of expression. Therefore, multiple reporters can be combined using appropriate FP spectral variants and orthogonal SynZip pairs (Figs 4 and 5). Based on the palette of available SynZips and FPs, we can envision to combine up to three dPSTRs in the same cell^12^,^19^. Note also that while it is feasible to estimate the nuclear enrichment only based on the fluorescent channel of the dPSTR and the whole-cell object, an exact quantification of the nuclear and cytoplasmic intensities will require a nuclear tag occupying one of the few fluorescent channels available for FP measurements.

Luminescence microscopy has also demonstrated the capability of recording the fast dynamics of protein expression^47^,^48^. However, due to the low photon flux generated by the luciferase, long integration times that can last several minutes are required. To achieve sub-minute temporal resolution, Mazo-Vargas *et al*.^47^ recorded a Z-stack with five planes with 10 s exposure for each plane. In microscopy experiments, a trade-off has to be reached between the frequency of acquisition, and the number of XY-stage positions visited. The longer the acquisition at each field of view lasts, the fewer XY-positions can be imaged. The parallel imaging of multiple positions can greatly improve the throughput of an experiment by allowing to increase the number of single-cell recordings, thereby improving the statistics of the measurements. To reach a 35 s time resolution, we have imaged three positions in three different wells recording close to 50 images per time point with more than 250 cells monitored for each well. The long exposure time required for luminescence data acquisition would clearly prevent reaching such imaging frequency and high number of cells, thereby lowering the resolution and statistical significance of the acquired data set.

The MS2 or PP7 technologies, which allow the detection of mRNA transcription in individual cells, offer complementary information to our reporter. We have implemented it for the detection of mRNA production arising from the p*STL1* promoter. The dynamics of the transcription site apparition indicate that there is roughly a 2 min delay between the production of the mRNA and appearance of the protein, during which protein synthesis is occurring. While the PP7 signal at the transcription site can provide rich information about the transcription dynamics, it has to be noted that its detection and automated quantification is not straightforward. PP7 measurements require the acquisition of Z-stacks with high magnification, thereby limiting the speed of acquisition and the size of the field of view, and hence, the number of cells that can be imaged. Moreover, the automated detection of the fluorescent signal at the transcription site is more complex than the measurement of the relocation of the dPSTR sensor from the cytoplasm to the nucleus of the cell.

We believe that our assay offers important advantages over current techniques. However, one limitation arises from the level of expression of the constitutive FP, which has to match to a certain extent the amount of the inducible binding partner (Supplementary Fig. 4). If the constitutive FP is expressed at too high levels, it becomes difficult to quantify small changes in protein expression. At the other extreme, low levels of FPs can lead to the saturation of the nuclear signal when all the FPs are bound to a 2xNLS-SZ moiety. In addition to providing important advantages in the quantification of the expression dynamics, the presence of the destabilizing sequence strongly reduces the chances of observing a saturation of the signal in the nucleus, since the 2xNLS-SZ peptide does not accumulate in the cell (Supplementary Fig. 5a–c). However, the sensitivity of the measurement is slightly decreased and therefore, when using the same threshold, fewer expressing cells are detected with the unstable version of the dPSTR (Fig. 4d). In this study, we used a mildly expressed promoter p*RPL24A* to control the FP abundance. We have tested another stronger constitutive promoter and have observed a lower sensitivity of this construct to detect weak expression at 0.1 M NaCl (Supplementary Fig. 4e).

The dPSTR technique applied to the *STL1* and *GPD1* promoters allowed to quantify events that could not be observed before with techniques based on FP expression. Our previous analysis of the p*STL1*-induced expression^11^ led us to postulate that stress responsive genes were induced with a large temporal variability due to slow chromatin remodelling events taking place at the induced locus. The real-time observation of protein synthesis with dPSTR allows now to confirm the large variability in dynamics and levels of p*STL1* expression. In comparison with a *GPD1* promoter, p*STL1* is induced with slower dynamics and larger intrinsic noise. Since there is basal expression of *GPD1* in unstressed cells, we believe that switch from a repressed to an active promoter via the chromatin remodelling step could be largely responsible for the striking kinetic differences measured between these two promoter elements. Interestingly, and somewhat in contradiction with our prediction, the correlation between the Hog1 signalling output as measured by the integral below relocation curve and the p*GPD1*-dPSTR output is not better than with p*STL1*. This implies that it is not only the chromatin-remodelling step that uncouples signalling and protein expression outputs. However, in the case of p*GPD1*, we cannot attribute this to a stochastic activation of the transcribed locus, since two p*GPD1* promoters in the same cells are strongly correlated. Therefore other extrinsic factors such as the amount of polymerase or the number of ribosomes could strongly influence the expression output independently of the Hog1 signal. It remains to be tested what parameters and how much of the kinase activity profile are encoded in the expression output.

More generally, this novel assay will now allow us to investigate the contribution of the various factors active at the promoters that control the kinetics of gene activation. Moreover, thanks to the destabilized nature of the reporter, it will become possible to study the processes implicated in the memory in successive stress events, such as the repositioning of the chromatin on the transcribed locus. In addition, due to the conservation of NLS sequences, this reporter could be easily adapted to quantify the dynamics of protein synthesis in higher eukaryotes.

## Methods

### Strains and plasmids

Yeast strains and plasmids are listed in Supplementary Tables 1 and 2. dPSTR plasmids were constructed by cloning different parts of the reporter into the single integration vectors pSIVU or pSIVL vector backbone (see below). The p*STL1*-dPSTR^R^ monitors expression arising from the promoter p*STL1*, and is based on an RFP variant (mCherry) (R for red), and the SynZips SZ1 and SZ2 (refs 18, 19). The p*STL1*-dPSTR^Y^ is based on the YFP variant mCitrine (A206K L221K) and the pair of SynZips SZ3 and SZ4. The FPs were expressed from the constitutive promoters p*RPL24A* or p*RPL15A*, cloned between SacI and XbaI. The FPs mCherry or mCitrine were cloned HindIII-SalI. The SynZips were cloned SalI-NheI. For the inducible part, a second MCS was designed and subcloned between AatII-SphI. The promoter p*STL1* (−800 to −1) (ref. 11) is cloned SacI-XbaI. The destabilization sequence UbiY is cloned XbaI-HindIII. To generate the p*GPD1*-dPSTRs, the p*STL1* promoter was replaced by p*GPD1* (−1,000 to −1) in all the constructs. The SynZips were cloned between SalI-NheI, and the *CYC1* or *SIF2* terminators XhoI-KpnI. We deposited at Addgene a set of plasmids for the dPSTR system, along with maps and sequences.

The plasmids were transformed in a yeast strain from a W303 background, bearing a Hta2-CFP nuclear marker (ySP37 (ref. 49)). Hog1 was tagged with mCitrine using the plasmid pKT139 (ref. 50).

To integrate both transcriptional units of the dPSTR, we developed a set of single integration vectors that entirely replace the selection marker gene by recombination with its promoter and terminator (Wosika *et al*. manuscript in preparation). The pSIV carries two MCSs, separated by the transcription unit that will compensate for the auxotrophy of the strain. The first MCS is constructed in the pSIV backbone, whereas the second MCS is cloned in a different intermediate vector, and subcloned into the pSIV between AatII and SphI. The pSIVL integrates into the *leu2* locus and carries a scrambled sequence of the *LEU2* gene, whereas the pSIVU integrates into the *ura3* locus and comprises the *Candida albicans URA3* gene, in order to avoid any undesirable recombination. The plasmids were transformed into our reference strain after digestion with PacI, separating the bacterial part from the yeast part of the plasmid.

For each transformation, 8–10 clones were screened based on their fluorescence intensities, and four clones with similar expression levels of the FP were further analysed by a time-lapse experiment upon stimulation with 0.2 M NaCl, to discard clones that would display an aberrant relocation behaviour.

### Sample preparation

The cells were grown overnight in synthetic medium to saturation (YNB: CYN3801, CSM: DCS0031, ForMedium). They were diluted to an OD_600_ of 0.05 in the morning and grown for at least 4 h before the start of the experiment. All the time-lapse experiments were performed in well slides, for which selected wells of 96-well-plates (MGB096-1-2LG, Matrical Bioscience) were coated with a filtered solution of Concanavalin A in H_2_O (0.5 mg ml^−1^, C2010-250MG, Sigma-Aldrich) for 30 min, rinsed with H_2_O and dried for at least ten hours. Before the experiment, the cells were diluted to an OD_600_ of 0.04, briefly sonicated, and 200 μl of cell suspension were added to a well. Imaging was started 30 min later to let cells settle to the bottom of the well. To stimulate the cells, 100 μl of a 3X stress solution was added in the well (see Supplementary Table 3 for the concentrations).

### Microscopy

Images were acquired on a fully automated inverted epi-fluorescence microscope (Ti-Eclipse, Nikon) controlled by micro-manager^51^ and placed in an incubation chamber set at 30 °C, with a × 40 oil objective and appropriate excitation and emission filters. The excitation is provided by a solid-state light source (SpectraX, Lumencor). The images were recorded with an sCMOS camera (Flash4.0, Hamamatsu). A motorized XY-stage allowed recording multiple fields of view at every time point. CFP (50 ms), RFP (300 ms) and YFP (150 ms for Hog1, 300 ms for dPSTR^*Y*^) and two bright-field (10 ms) images were recorded at time intervals varying from 35 s to 5 min.

### Data analysis

Time-lapse movies were analysed with the YeastQuant platform (Supplementary Fig. 2)^27^. The nuclei of the cells were segmented by thresholding of the CFP images. The contour of the cell around each nucleus was detected using two bright-field images. The cytoplasm object was obtained by removing the nucleus object expanded by two pixels from the cell object. Dedicated scripts in Matlab (The Mathworks) were written to further analyse the data. Only cells tracked from the beginning to the end of the movie were taken into consideration. In addition, a quality control was applied on each trace and only the traces with low variability in nuclear and cell area, and nuclear CFP fluorescence were kept for further analysis (typically more than 65% of the tracked cells). The curves displayed in the figures represent the mean and s.e.m. of these selected traces for one representative experiment out of three true biological replicates.

For each cell, the difference between its average intensity in the nucleus and in the cytoplasm was calculated at every time point to plot the nuclear enrichment of the dPSTR and of the Venus. The basal level is calculated as the mean of the two time points immediately following the addition of NaCl (*T*=0 min and *T*=2 min), in order to take in account the sudden increase in fluorescence intensity triggered by the abrupt nuclear enrichment upon shrinking of the cells when NaCl is added. This is an artefact of the measurement and not a transcriptional response of the cell (Supplementary Fig. 1). The maximal enrichment was obtained for each single-cell trace smoothed by a moving average of three points. For each single cell, the corrected nuclear enrichment of the dPSTR was calculated as the smoothed trace subtracted by its basal level. The expression output represents the maximal corrected nuclear enrichment of the dPSTR. The expression thresholds were determined based on the expression outputs of the non-induced populations. The time to overcome the expression threshold was defined as the first time point when the corrected nuclear enrichment of the dPSTR is equal to or greater than the expression threshold. Finally, the time to reach half of the expression output was extracted from each non-smoothed trace as the first time point at which the nuclear enrichment is equal or grater than half of the maximal nuclear enrichment. The intrinsic and extrinsic noise were calculated according to the formula from Elowitz *et al*.^7^:


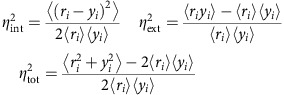


since: 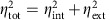 we plot the fraction of the intrinsic noise as: 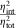. *r*_*i*_ and *y*_*i*_ are the normalized nuclear accumulation from the *i*^th^ cell in the red and yellow channels, respectively. The normalization factors were obtained from the highest and lowest average intensity from the entire data set for one replicate.

Hog1 nuclear accumulation was calculated as the ratio of the nuclear over the cytoplasmic intensities. The signalling output of each single cell was calculated as the area under Hog1 curve during a time interval determined as the period of Hog1 activity for the average population.

### mRNA transcription sites measurements

The measurement of mRNA transcription sites was performed using the PP7 technique^31^. The PP7-2xGFP under the control of the p*MET* promoter was cloned in an integrative vector pRS304 integrated in the TRP1 locus. The original promoter p*POL1* from vector pDZ306 (Addgene# 35196) was replaced by the promoter p*STL1* and drives the transcription of 24 stem loops, which can be tightly bound by a dimer of PP7. The loops were integrated in the *GLT1* locus to generate a very long transcript of 6.4 kb to facilitate the visualization of the PP7 foci. Cells were imaged with the same set-up as described above. A × 60 objective and piezo Z-stage (Nano-Z200, Mad City Labs) was used and 3 Z-stacks from -2 to +2 μm were acquired every 30 s. For the identification of the nucleus, an Hta2-mCherry tag was present in the strain and imaged with similar Z-stacks. Two bright-field images were also recorded for segmentation of the cells. The maximum intensity projections of the GFP and RFP images were used in the YeastQuant pipeline. As a measurement of the transcription site intensity, the difference between the average intensity of the 20 brightest pixels in the nucleus and the average intensity of the nucleus is calculated.

### Flow Cytometry

The flow cytometry experiments were performed as previously described in ref. 30. Briefly, a cell population was induced by NaCl and samples were taken at different time points and immediately blocked with cycloheximide (0.1 mg ml^−1^). Cells were incubated for at least 2 h to allow the maturation of the FP before being measured by flow cytometery (FACSCalibur, BD). Ten thousand events were measured and a gating was applied on the forward and side scattering to discard clusters of multiple cells.

## Additional information

**How to cite this article:** Aymoz, D. *et al*. Real-time quantification of protein expression at the single-cell level via dynamic protein synthesis translocation reporters. *Nat. Commun.* 7:11304 doi: 10.1038/ncomms11304 (2016).

## Supplementary Material

Supplementary Information Supplementary Figures 1-10, Supplementary Tables 1-4, Supplementary Note 1 and Supplementary References
