# Supplementary material for: Real-time quantification of protein expression at the single-cell level via dynamic protein synthesis translocation reporters
Source: Nat Commun. 2016 Apr 21;7:11304. doi: 10.1038/ncomms11304 (PMC4844680; doi:10.1038/ncomms11304)
Supplement: Supplementary Information — Supplementary Figures 1-10, Supplementary Tables 1-4, Supplementary Note 1 and Supplementary References [file ncomms11304-s1.pdf]

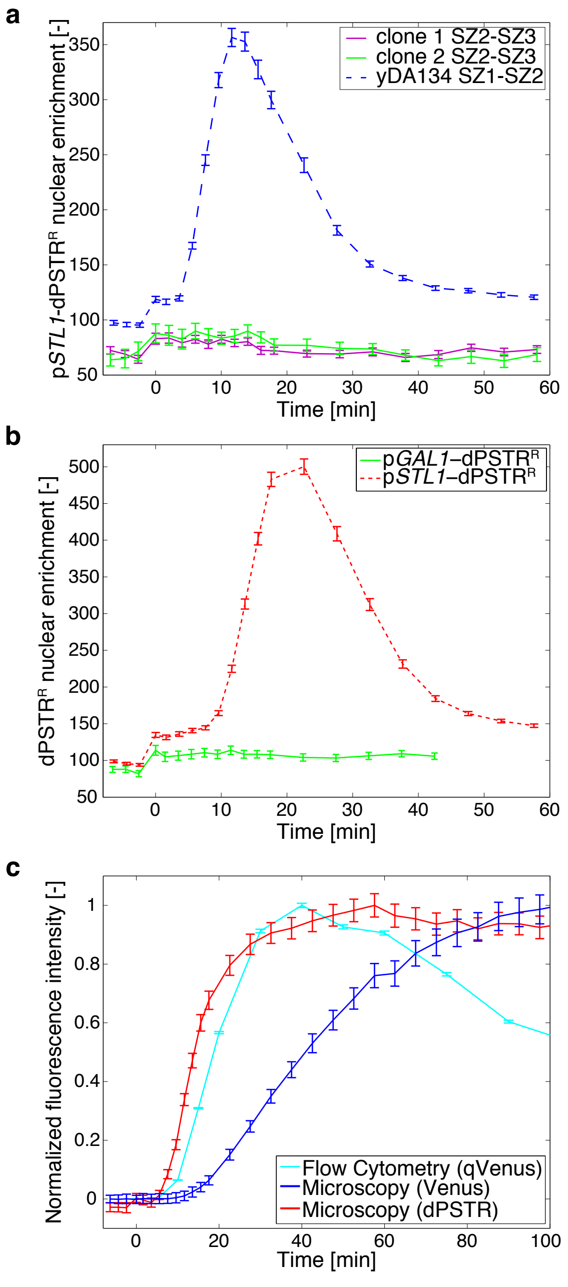

Supplementary Figure 1: Control experiments for the dPSTR.

a. Stimulation with 0.2 M NaCl of two different clones carrying a p*STL1*-dPSTR^{R2,3} containing the SynZips SZ2 and SZ3, which are not interacting. The non-functional p*STL1*-dPSTR^{R2,3} does not display any nuclear enrichment. For comparison, the functional p*STL1*-dPSTR^{R2,1} (yDA134) is plotted with a dashed blue line.

b. Stimulation with 0.4 M NaCl of a strain carrying a p*GAL1*-dPSTR^{R}, which is not expressing upon hyperosmotic shock, and does not display any nuclear enrichment. For comparison, the p*STL1*-dPSTR^{R} (yDA134) is plotted with a dashed red line.

c. Comparison of p*STL1* expression induced by 0.2 M NaCl measured by flow cytometry (p*STL1*-qVenus, cyan, N_C~10'000) or microscopy for Venus expression or p*STL1*-dPSTR^{R} relocation (blue and red respectively, data from **Fig. 1**). Note the close overlap between the rise of transcription quantified with flow cytometry or the dPSTR. To allow for a direct comparison of the three methods, the fluorescent values measured are normalized.

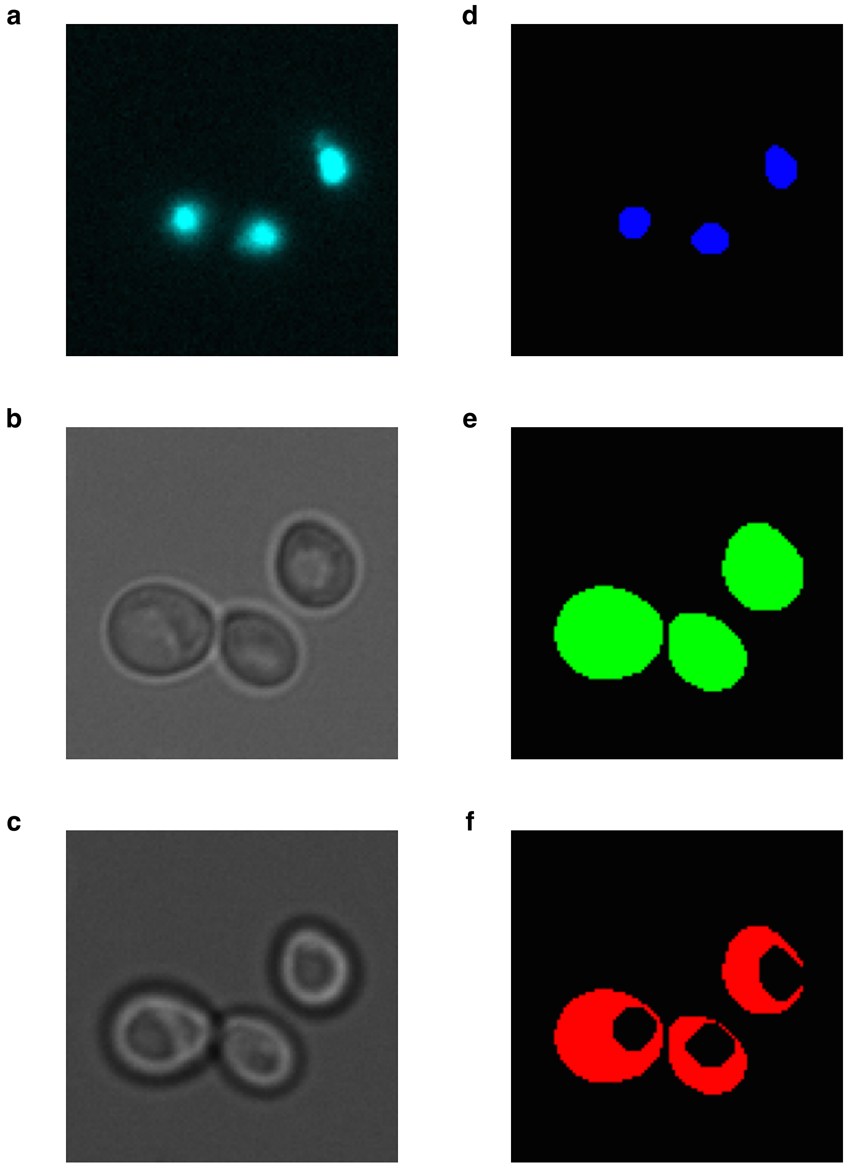

Supplementary Figure 2: Image segmentation process using YeastQuant
a. - b. - c. Microscopy images used for the segmentation: histone tag CFP (**a**), bright field image in the focal plane (**b**), and out of focus bright field image (**c**, z=-2.5µm).
d. - e. - f. Different objects are defined by the segmentation process. First, the Nucleus is characterized using the CFP image (**d**). The two bright field images are used to find the cell contour and define the Cell object (**e**). Then, the Nucleus object, enlarged by 2 pixels, is subtracted to the Cell object, to define the Cytoplasm object (**f**).

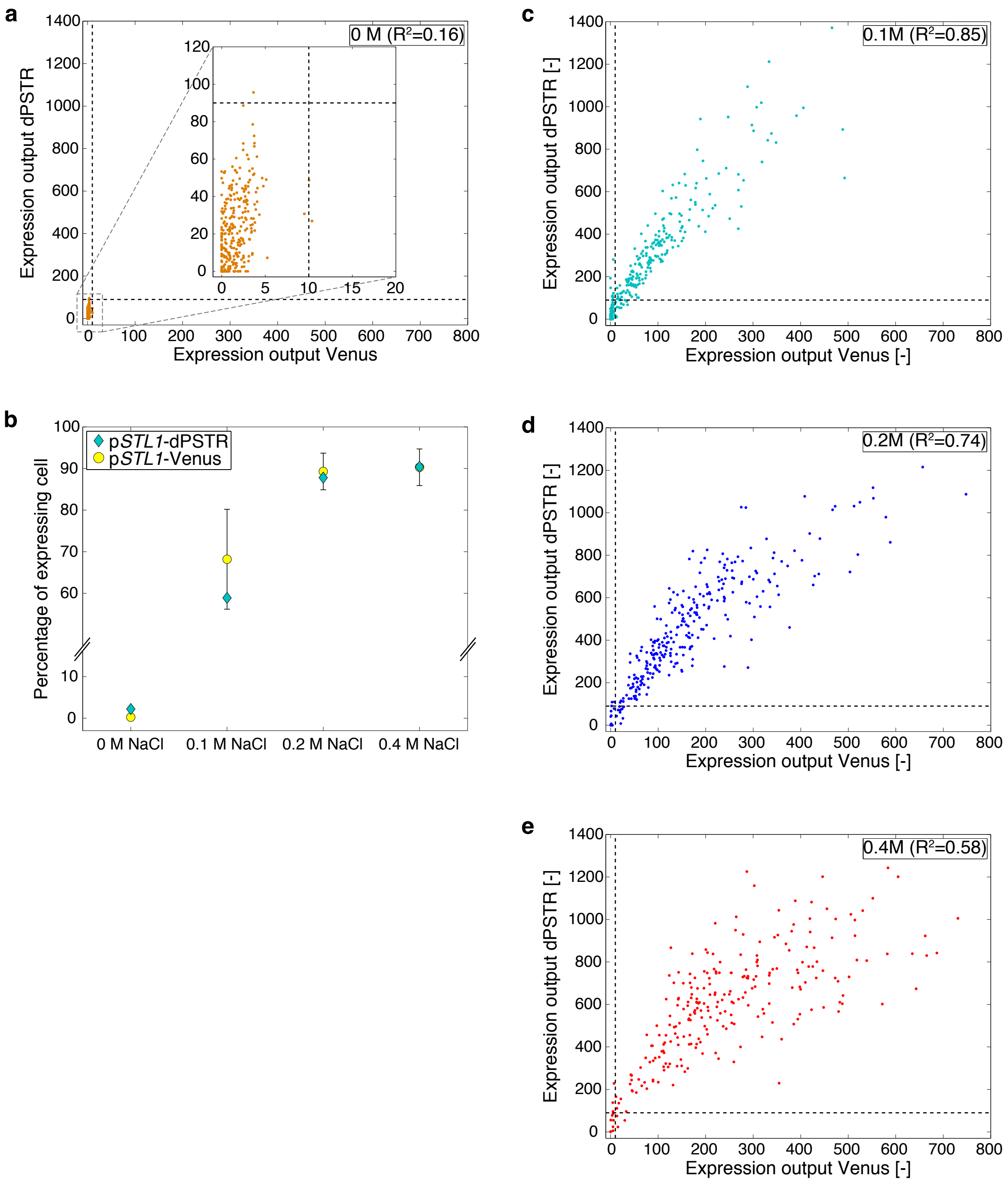

Supplementary Figure 3: Comparison of the level of expression and the dynamics of expression measured by the dPSTR and by the Venus fluorescence apparition.

a. - c. -d. - e. Single cell correlation of the expression output measured by the dPSTR or the Venus, for the non-induced control 0 M NaCl (**d**) and each concentration: 0.1 M NaCl (**c**), 0.2 M NaCl (**d**), 0.4 M NaCl (**e**). The dashed lines represent the thresholds for expression.

b. Percentage of expressing cells measured by the dPSTR or by the promoter-FP method, for each concentration. Cells having an expression output above the expression threshold plotted in **a**, c, **d** and **e** are defined as expressing cells. Note that the proportions are similar using the two methods. Each dot represents the mean of three experiments and the error bars, the standard deviations.

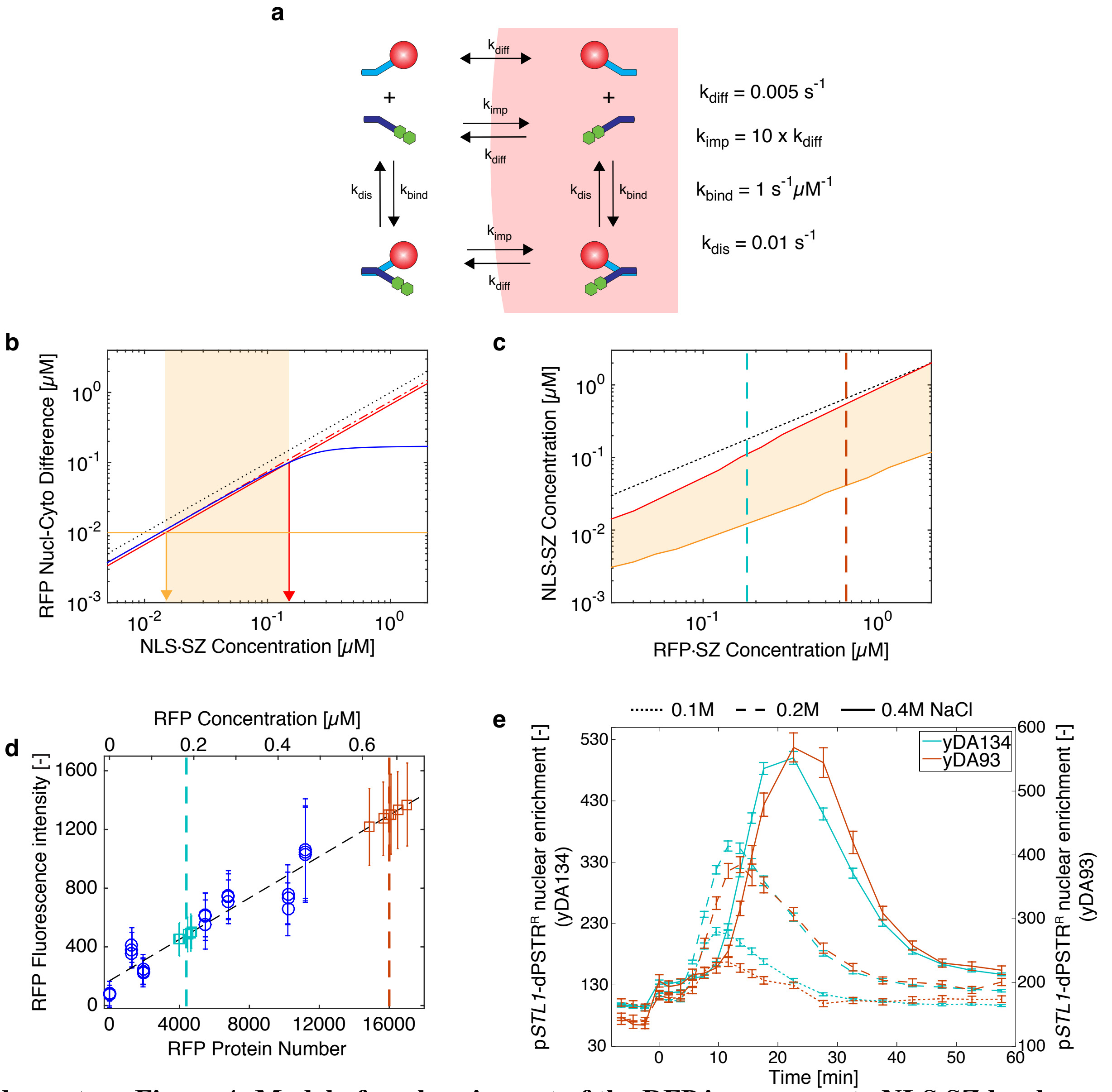

Supplementary Figure 4: Model of nuclear import of the RFP in response to NLS·SZ levels.

a. Schematic of the reactions implemented in the model and their corresponding rate constants. See Supplementary Note for further details.

b. Difference between nuclear and cytoplasmic RFP concentrations expected for a fixed total RFP·SZ concentration (0.2 µM) and a range of NLS·SZ concentration (blue line). The dotted black line indicates the total NLS·SZ concentration. At low concentrations, there is a linear relationship between NLS·SZ level and nuclear minus cytoplasmic RFP concentration (red dash-dotted line). At higher concentration, the nuclear enrichment of the RFP saturates since an increasingly higher fraction of the RFP is already localized in the nucleus. If we tolerate 10% deviation from linearity (solid red curve), we obtain an upper limit for the concentration of NLS·SZ that can be measured (red arrow). The sensitivity of the detection allows to observe a 10% increase in RFP nuclear enrichment, thereby setting a lower limit to the concentration of NLS·SZ that can be measured (orange arrow). Therefore, the shaded area represents the sensitivity range of the assay for the selected RFP·SZ expression level.

c. The lower and upper limits of sensitivity of the system are calculated for a range of RFP·SZ concentrations. An NLS·SZ sensitivity window is depicted with a shaded area bound by lower and higher limits (orange and red lines, respectively). The dotted black line indicates the case where NLS·SZ = RFP·SZ.

d. Calibration of the protein number and concentration of RFP·SZ in the reporter strains (yDA134, blue squares and yDA93 orange, 6 biological replicates) relative to endogenously tagged proteins with different expression levels (blue circles, three biological replicates, see Supplementary Table 4). The mean and standard deviation of more than 500 single cells is plotted and fitted by linear regression (dashed black line). The expression level of RFP is found to be 4400±300 protein per cell or 0.18µM for yDA134 and 16'000±750 protein per cell or 0.66µM for yDA93 (dashed line in panel c and d).

e. Normalized nuclear enrichment in course of time for yDA134 (blue) and yDA93 (orange) stimulated with 0.1 (resp. N_C=558 and N_C=341), 0.2 (resp. N_C=655 and N_C=297), or 0.4M NaCl (resp. N_C=802 and N_C=268). Note the good overlap between the responses of the two strains at 0.2 and 0.4M NaCl and the lower sensitivity at 0.1 M.

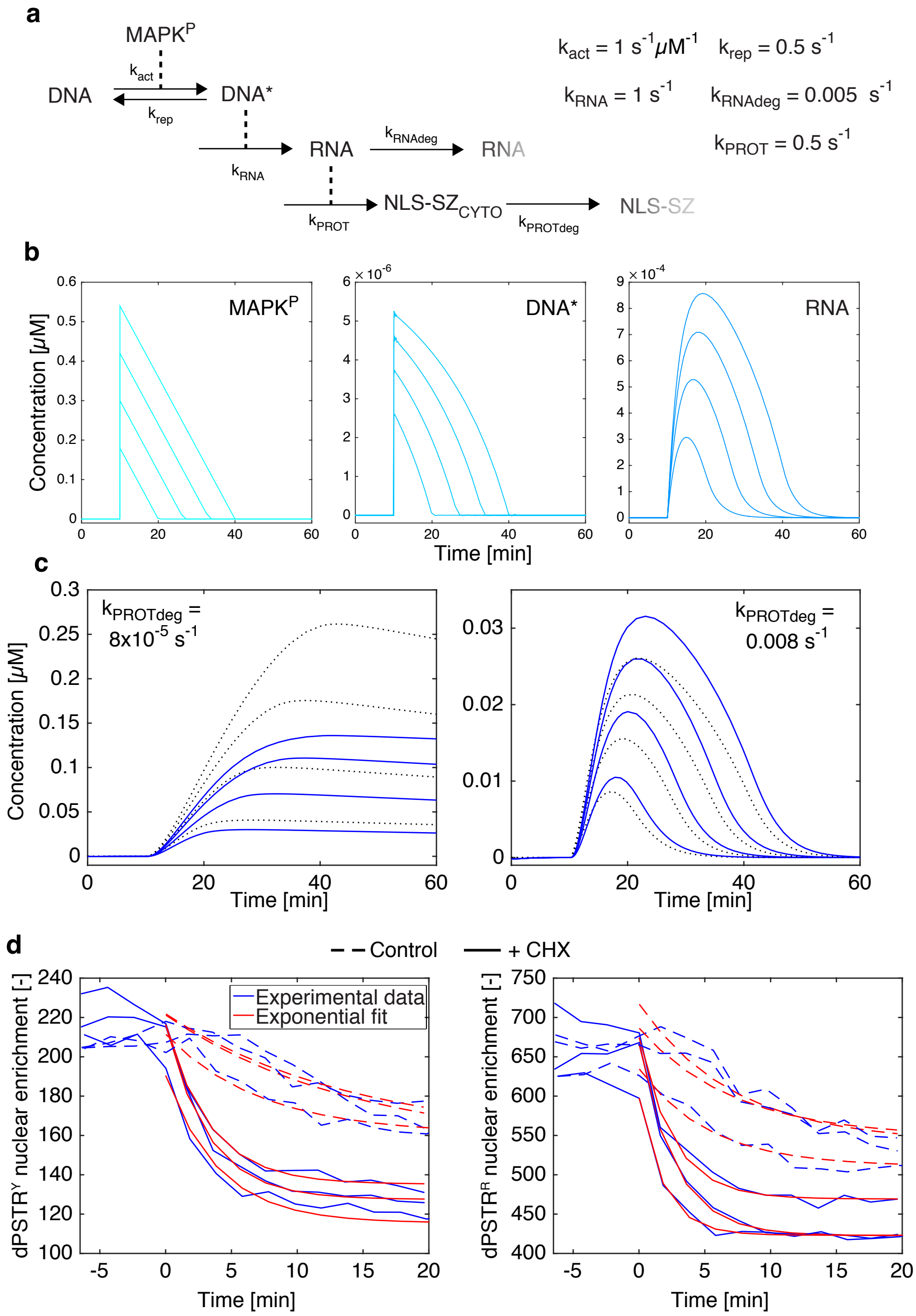

Supplementary Figure 5: Model of NLS·SZ synthesis in presence or absence of degradation

a. Schematic of a simple expression model based on mass-action kinetics for the synthesis of the NLS·SZ. The NLS·SZ is produced in the cytoplasm and enters in equilibrium with the RFP·SZ as described in Supplementary Figure 4. See also Suplementary Note.

b. Dynamics of the $MAPK^P$ which is the input to the model and the resulting gene activation DNA* and RNA production.

c. The dynamics of total NLS·SZ production (dotted line) is compared to the RFP nuclear-cytoplasmic enrichment (solid line) in the case where the NLS·SZ is stable or if it has a half-life of 2 min. From this model, it can be observed that the dynamics of RFP nuclear enrichment can be limited by two reactions, the formation of the complex between NLS·SZ and the RFP·SZ and the import of the NLS-SZ in the nucleus. Both of these reactions happen with fast dynamics thus allowing a monitoring of the protein production in the sub-minute time scale. The deactivation of the system depends on three reactions, the repression of the active gene, the degradation of the mRNA and the degradation of the NLS-SZ. In our experimental data, the return of the dPSTR nuclear enrichment to pre-stimulus level takes place on the order of 10 minutes suggesting that the NLS·SZ degradation is not the limitting factor for this process.

d. Characterization of destabilization sequence half-life. Cells bearing the p*GPD1*-$dPSTR^Y$ and p*GPD1*-$dPSTR^R$ were grown overnight in SD+1M sorbitol to increase the basal level of p*GPD1* expression (note the differences in nuclear enrichment before time 0 compared to Fig. 5b). Cells were treated with cycloheximide (solid line, N_C = 380) or not (dashed line, N_C = 383). A half-life for the UbiY-NLS-SZ is estimated to 2.1±0.5 min (3 experiments, mean and standard deviation of the RFP and YFP dynamics) based on an exponential fit of the data (red line).

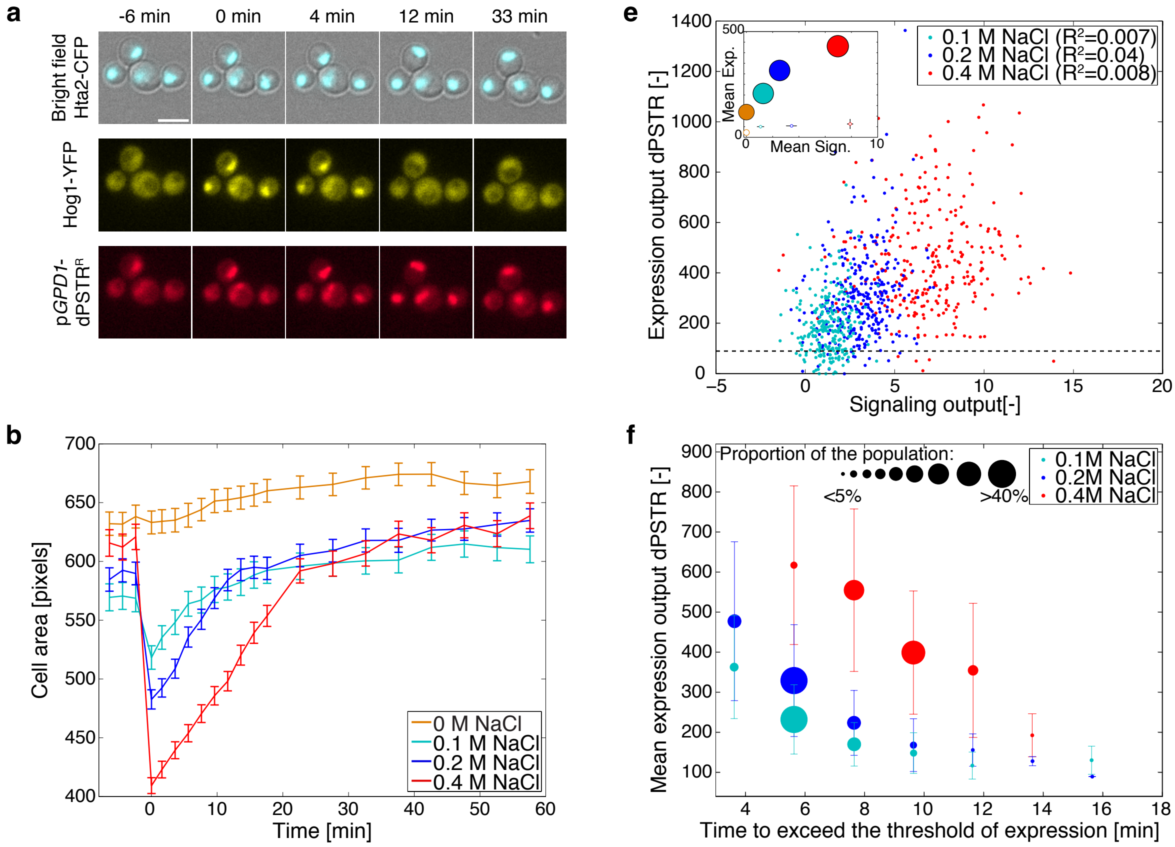

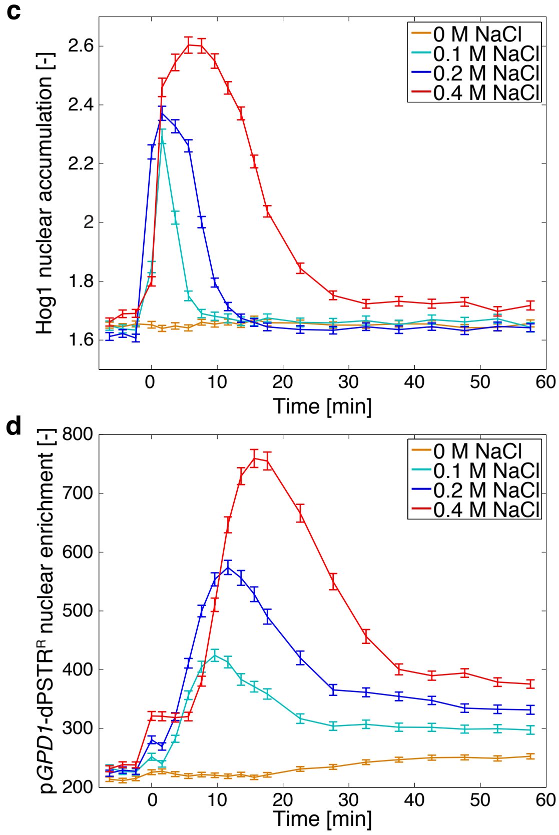

Supplementary Figure 6: Lack of correlation between Hog1 activity and p*GPD1* expression at the single cell level.

a. Microscopy images of a strain with Hog1 tagged with mCitrine and carrying the unstable p*GPD1*-dPSTR^R stimulated with 0.2 M NaCl. The nuclear accumulation of Hog1 precedes protein expression. Scale bar represents 5µm.

b. - c. - d. Quantification of the cell area (**b**), Hog1 nuclear accumulation measured as the ratio between nuclear and cytoplasmic fluorescence (**c**) and p*GPD1*-dPSTR^R nuclear enrichment (**d**) for cell population stimulated with 0 (orange, N_C=430), 0.1 (cyan, N_C=250), 0.2 (blue, N_C=319) and 0.4 M NaCl (red, N_C=279).

e. Scatter plot of the signaling output measured as the integral below the Hog1 nuclear accumulation curve versus the expression output measured by p*GPD1*-dPSTR^R. The dashed line represents the threshold for expression. Inset represents the correlation between the mean signaling output (x axis) and the mean expression output (y axis) for the expressing cells (filled circles, R^2=0.91) and the non expressing cells (empty circles, R^2=0.66). The size of the marker is indicative of the percentage of cells in each category.

f. Correlation between the time needed to overcome the expression threshold and the expression output. The mean expression output and the standard deviation were calculated for groups of cells which exceed the expression threshold at the same time point. The marker size is indicative of the percentage of cells (from the total population) in each group. Note that the marker size is bigger than in Figure 3, indicating that a higher proportion of the population expresses p*GPD1* compared to p*STL1*.

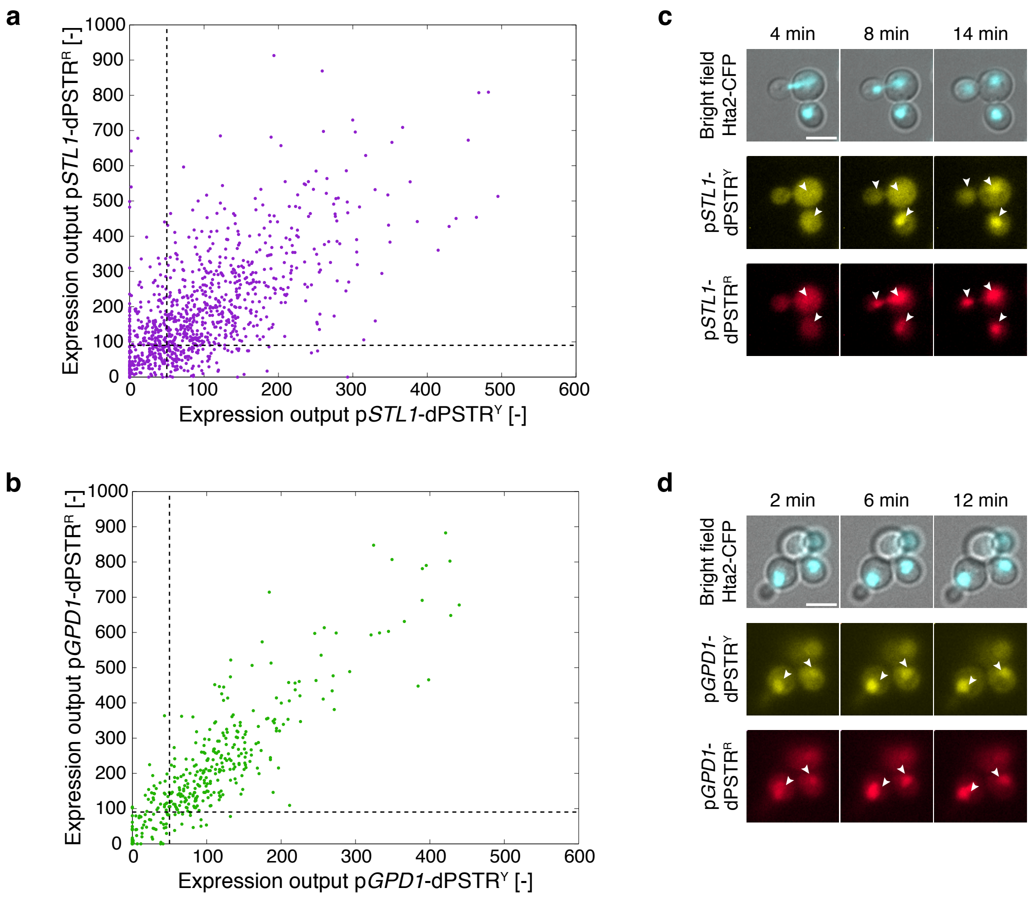

Supplementary Figure 7: Expression output of cells containing double p*STL1* or p*GPD1* dPSTRs.

a. - b. Expression output measured for cells from **Figure 5** expressing two p*STL1*-dPSTRs (**a**) or two p*GPD1*-dPSTRs (**b**).

c. - d. Microscopy images of cells bearing an Hta2-CFP and expressing two p*STL1*-dPSTRs (**c**) or two p*GPD1*-dPSTRs (**d**). The white arrowheads show nuclei in the focal plane. Scale bars represent 5µm.

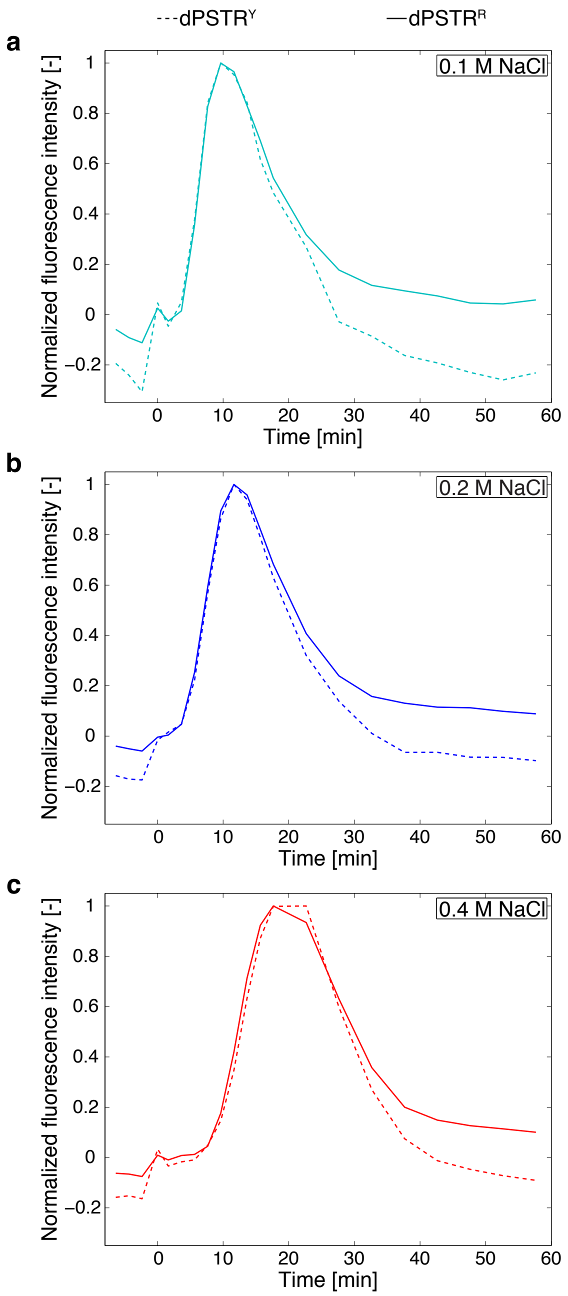

Supplementary Figure 8: Control experiments for the p*STL1*-dPSTR^{R} and p*STL1*-dPSTR^{Y} sensors. **a. - b. - c.** Normalized traces of p*STL1*-dPSTR^{Y} (dashed line) and p*STL1*-dPSTR^{R} (filled line) for cells stimulated with 0.1 M NaCl (**a**), 0.2 M NaCl (**b**) and 0.4 M NaCl (**c**). The weaker overlap observed at later time-points could be due to a differential bleaching of the two fluorescent proteins.

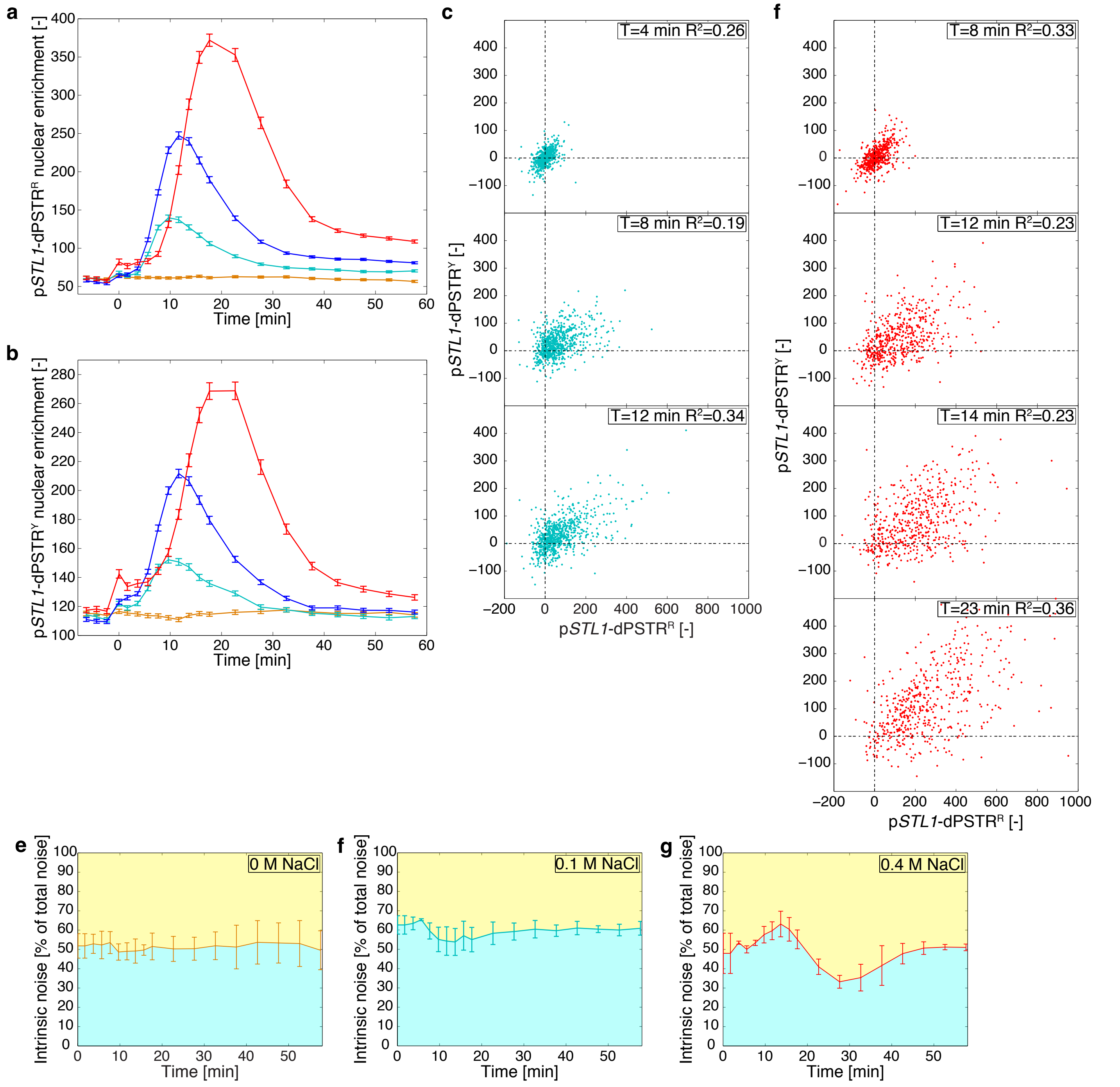

Supplementary Figure 9: Dynamic quantification of the expression noise of p*STL1* with different stimuli.

a. - b. Quantification of p*STL1*-dPSTR^R (**a**) and p*STL1*-dPSTR^Y nuclear enrichment (**b**) for cell population stimulated with 0 (orange, N_C=670), 0.1 (cyan, N_C=765), 0.2 (blue, N_C=958) and 0.4 M NaCl (red, N_C=530). The 0.2M induction was used in **Figure 5**.

c. Single cell correlation between the corrected nuclear enrichment of each p*STL1*-dPSTR nuclear enrichment at given times, for cells stimulated with 0.1M NaCl: T=4 min, T=8 min, T=12 min.

d. Same as above for cells stimulated with 0.4M NaCl at times: T=8 min, T=12 min, T=14 min, T=23 min.

e. - f. - g. Evolution of the intrinsic noise for unstimulated cells (**e**) or cell stimulated with 0.1 M NaCl (**f**) or 0.4 M NaCl (**k**). The mean of 3 biological replicate with the SD is ploted. Note the transient rise in intrinsic noise observed at the onset of gene expression.

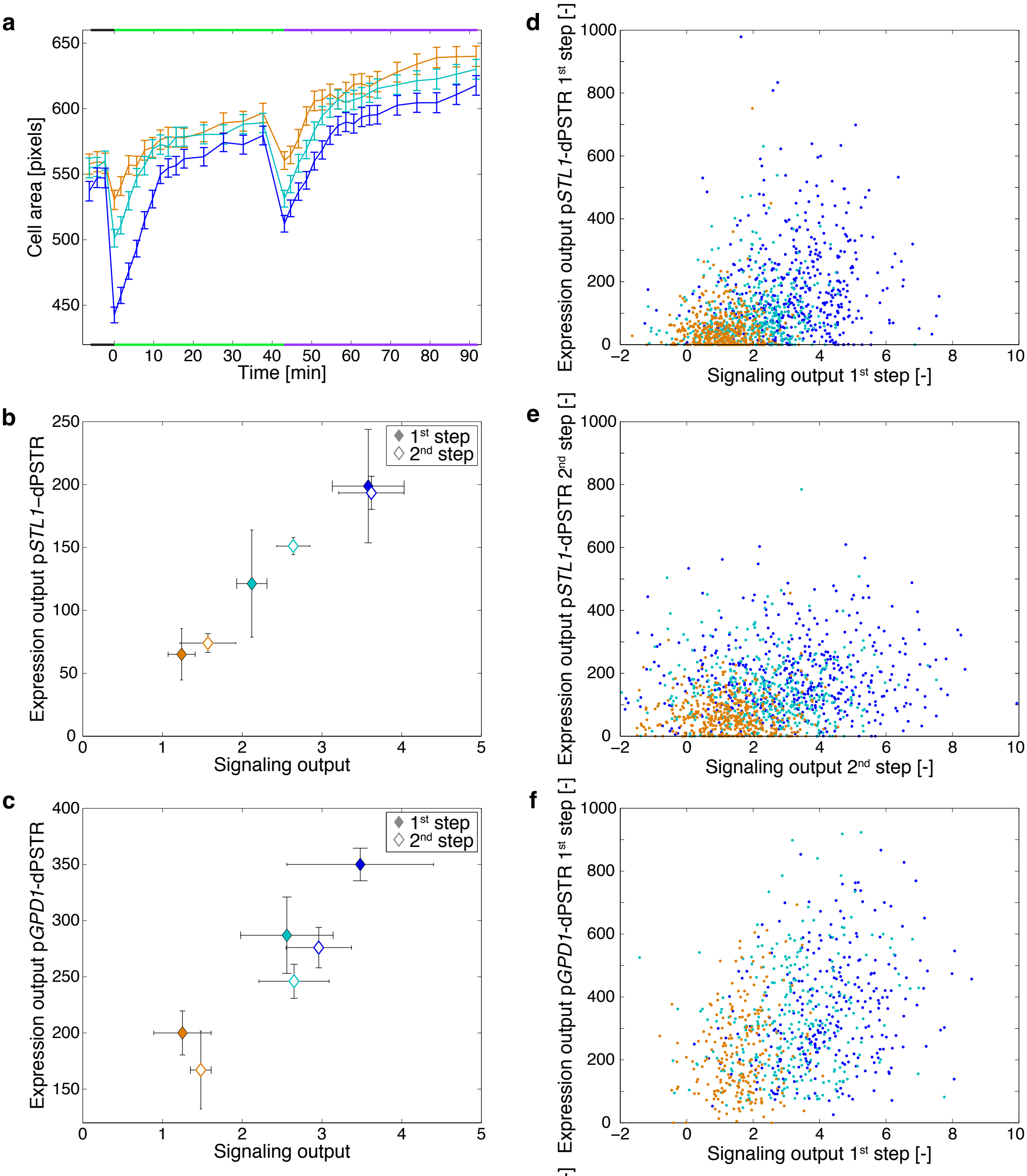

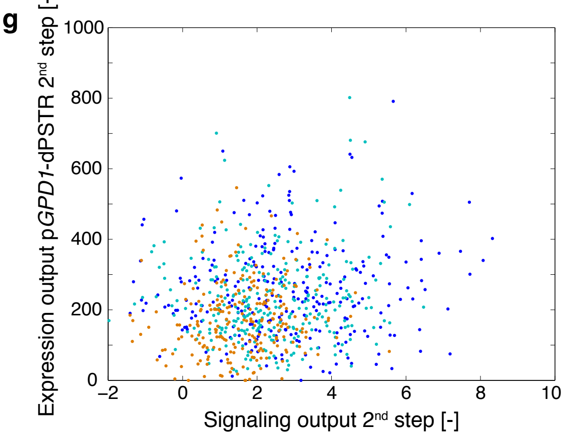

Supplementary Figure 10: Correlation between Hog1 activity and protein expression in two consecutive stimulations.

a. Quantification of the cell area for the cells shown in **Figure 6b**: 0.1 → 0.2 (orange), 0.15 → 0.3 (cyan) and 0.2 → 0.4 (blue).

b. - c. Population correlation between the expression output and the signaling output for the first step (filled diamonds, R^2=0.99) and second step (open diamonds, R^2=0.98) for p*STL1*-dPSTR (**b**) or p*GPD1*-dPSTR (**c**) (1^{st}step R^2=0.99 and 2nd step R^2=0.99) . Each point represents the average of three biological replicates. Error bars are the standard deviations.

d. - g. Single-cell correlation between the expression output and the signaling output for the 1st step (**d** and **f,** data from **Figure 6b** and **c**) and the 2nd step (**e** and **g,** data from **Figure 6d**). For all the correlations, $R^2<10^{-3}$.

Supplementary Tables

Supplementary Table 1: List of yeast strains used in this study

Strain	Background	Genotype	Plasmid	Figure
ySP2 [1]	W303	*MATa leu2-3,112 trp1-1 can1-100 ura3-1 ade2-1 his3-11,15*		
ySP37	W303	*HTA2-CFP*		
yDA119	YSP37	*HTA2-CFP*<br>*ura3: pSTL1-Venus-*$dPSTR^{R2-1}$ (*)	 pDA176	1, S1c, S3
ySP9	W303	*leu2: pSTL1-quadrupleVenus*	pSP34	S1c
ySP374	ySP37	*ura3: HTA2-mCherry*<br>*trp1: PP7-GFP*<br>*glt1: his3 pSTL1-24xPP7-SL*	 pSP268<br>pSP264	2a, 2b
yDA134	ySP37	*HTA2-CFP*<br>*ura3: pSTL1-*$dPSTR^{R2-1}$<br>*HOG1-mCitrine: HIS3*	 pDA183	2b, 3, 6b, c, e and f, S1a and b, S4, S10a,b, d and e
yDA137	ySP37	*HTA2-CFP*<br>*ura3: pSTL1-*$dPSTR^{R2-1}$<br>*leu2: pSTL1-*$dPSTR^{Y4-3}$	 pDA183<br>pDA199	5a, d and e, S7a and c, S8, S9
yDA139	ySP37	*HTA2-CFP*<br>*ura3: pGPD1-*$dPSTR^{R2-1}$<br>*leu2: pGPD1-*$dPSTR^{Y3-4}$	 pDA193<br>pDA200	5, S5d, S7b and d
yDA155	ySP37	*HTA2-CFP*<br>*ura3: pGPD1-*$dPSTR^{R2-1}$<br>*HOG1-mCitrine: HIS3*	 pDA193	6d and g, S6, S10c, f and g
yDA156	ySP37	*HTA2-CFP*<br>*ura3: pGPD1-*$dPSTR^{R2-1}$<br>*leu2: pSTL1-*$dPSTR^{Y3-4}$	 pDA193<br>pDA199	4
yDA93	ySP37	*HTA2-CFP*<br>*ura3: pSTL1-*$dPSTR^{R2-1}$<br>*HOG1-mCitrine: HIS3*	 pDA140	S4d and e
yDA85	ySP37	*HTA2-CFP*<br>*ura3: pSTL1-*$dPSTR^{R2-3}$	pDA137	S1a
yDA112	ySP37	*HTA2-CFP*<br>*ura3: pGAL1-*$dPSTR^{R2-1}$	 pDA169	S1b, S5d

* The numbers in the superscript $dPSTR^{R2-1}$ indicate the pair of SynZip used.

Supplementary Table 2: List of plasmids used in this study

Plasmid	MCS1	MCS2	Backbone	Strain
pDA137	p*RPL24A*-mCherry-SynZip2	p*STL1*-UbiY-2xSv40NLS-SynZip3-t*CYC1*	pSIVU	yDA85
pDA140	p*RPL15A*-mCherry-SynZip2	p*STL1*-UbiY-2xSv40NLS-SynZip1-t*CYC1*	pSIVU	yDA93
pDA169	p*RPL24A*-mCherry-SynZip2	p*GAL1*-UbiY-2xSv40NLS-SynZip1-t*CYC1*	pSIVU	yDA112
pDA176	p*RPL24A*-mCherry-SynZip2	p*STL1*- 2xSv40NLS-Venus-SynZip1-t*CYC1*	pSIVU	yDA119
pDA183	p*RPL24A*-mCherry-SynZip2-t*SIF2*	p*STL1*-UbiY-2xSv40NLS-SynZip1-t*CYC1*	pSIVU	yDA134, yDA137
pDA193	p*RPL24A*-mCherry-SynZip2-t*SIF2*	p*GPD1*-UbiY-2xSv40NLS-SynZip1-t*CYC1*	pSIVU	yDA139, yDA155, yDA156
pDA199	p*RPL24B*-MCitrine-SynZip4-t*NUP53*	p*STL1*-UbiY-2xSv40NLS-SynZip3-t*CYC1*	pSIVL	yDA137
pDA200	p*RPL24B*-MCitrine-SynZip4-t*NUP53*	p*GPD1*-UbiY-2xSv40NLS-SynZip3-t*CYC1*	pSIVL	yDA139
pSP264	pGLT1-pSTL1-24xPP7StemLoop	-	pDZ306 Addgene:35196	ySP374
pSP34	p*STL1*-quadrupleVenus	-	pRS305	ySP9
pSP268	p*MET*-PP7-2xGFP	-	pRS304	ySP374

Supplementary Table 3: Concentrations of inducers

Final concentration	Concentration of inducer solution	Figure	Final concentration after second stress	Concentration of inducer solution	Figure
0.1 M	0.3 M	1, 3, 6 S2, S3, S4, S6, S8, S9	0.2 M	0.5 M	6, S10
0.15 M	0.45 M	6, S10	0.3 M	0.75 M	6, S10
0.2 M	0.6 M	1, 2, 3, 4, 5, 6, S1, S3, S4,, S6, S7, S8, S9	0.4 M	1 M	6, S10
0.4 M	1.2 M	1, 3,S1, S3, S4, S6, S8, S9			

Supplementary Table 4: List of yeast strains used for fluorescence microscopy calibration
Genotype: W303, *Protein-mCherry:Ura3*

Strain	Protein tag	Protein Number
ySP37*	-	0
ySP408	Ygr117c	1280
ySP426*,§	Ste12	1920
ySP534*	Kss1	5480
ySP399	Hog1	6'780
ySP400	Apt1	11'200
ySP405	Tda1	10'200

*These strains also bear a Hta2-CFP tag
§ Protein-mCherry:His

Supplementary Note 1

dPSTR relocation model
We have used a simplified model to estimate the amount of nuclear enrichment of the fluorescent protein as function of the expression of the inducible peptide (Supplementary Figure 4). The model contains three species: the fluorescent protein linked to the SynZip (FP·SZ), the inducible peptide (NLS·SZ) and the complex formed between those two proteins via the SynZip (FP·SZ·NLS). The K_d of the complex formed has been estimated to 10μM [2,3]. These three species are partitioned between two compartments: the nucleus and the cytoplasm. We estimate a passive diffusion of the FP·SZ into and out of the nucleus to be 0.005 s^{-1} [4]. Based on the level of nuclear enrichment of the Venus·SZ (Figure 1d), we estimate that the double NLS present on the NLS·SZ peptide allows at least a 10-fold enrichment of the proteins in the nucleus. Using these parameters, we have simulated the steady-state nuclear and cytoplasmic concentrations for each component for a range of total NLS·SZ and FP·SZ. For each condition, the nuclear to cytoplasmic difference is calculated as:

$$\Delta Nucl - Cyto = ([FP \cdot SZ_N] + [FP \cdot SZ \cdot NLS_N]) - ([FP \cdot SZ_C] + [FP \cdot SZ \cdot NLS_C])$$

Figure S4b displays the outcome of the model for a concentration of 0.2 μM of RFP·SZ. For low concentrations of the NLS·SZ, there is a linear correlation between nuclear enrichment of the RFP and the NLS·SZ concentration. However, there is a limit to the detection ability of the microscope and we estimate that only a 10% enrichment can be reliably detected at the single cell level (~10^{-2} μM), thereby setting a lower limit to the NLS·SZ that can be measured with the dPSTR (1.5×10^{-2} μM). If the NLS·SZ concentration reaches too high levels, the linear relationship with RFP nuclear enrichment is lost, as can be seen from the saturation of the blue curve in Supplementary Figure 4b. Allowing for 10% error in linearity between NLS·SZ level and nuclear enrichment measurement results in an upper limit in NLS·SZ that can be reached before saturation (1.6×10^{-1} μM). These upper and lower limits were calculated for a range of RFP·SZ concentrations (Supplementary Figure 4c).

In parallel to this modeling effort, we have quantified the level of RFP·SZ expression in the dPSTR strain. We measured a calibration curve based on the fluorescence intensity of mCherry tagged proteins with known expression levels [5, 6, 7]. After linear regression between protein numbers and fluorescent intensities, we can get a good estimate of the protein number of the constitutively expressed fluorescent protein in the dPSTR.

At 4 400 proteins per cell or 0.18 μM (for a 40 fl volume [8,9]), we can estimate the low and high limits of expression that can be quantified with the dPSTR between $1.2 \cdot 10^{-2}$ to $1.2 \cdot 10^{-1}$ μM of synthesized NLS·SZ. For comparison, we also quantified the fluorescence of another dPSTR strain with higher expression levels (16'000 prot. per cell, yDA93). The concentration range that can be quantified varies from $3.9 \cdot 10^{-2}$ to $6 \cdot 10^{-1}$ μM. These two strains have different sensitivity windows. As predicted by the model, we observe with this strain a lower sensitivity at detecting protein expression from the p*STL1* promoter at 0.1 M NaCl (Supplementary Figure 4e).

Model of NLS·SZ synthesis and degradation

We further developed this model to include a minimal transcriptional model in order to simulate the dynamics and level of nuclear import with a stable and unstable dPSTR (Supplementary Figure 5). The reactions and rate constants used in the model are presented in Supplementary Figure 5a. Briefly, the input to the model is the level of activated MAPK ($MAPK^P$), which increases after 10 min of simulation and declines in a linear fashion to reach zero after a time that is function of the level of activity upon stimulation. This mimics the different temporal windows of Hog1 activity upon various hyper-osmotic stresses. The active kinase turns the promoter into an active state. From this state, mRNA can be produced, which ultimately leads to the expression of the NSL·SZ. This peptide is produced in the cytoplasm, and it can bind to the FP·SZ and accumulate in the nucleus following the same reactions as described above. Once kinase activity returns to pre-stress levels, the promoting region of the DNA returns to its off-state, the mRNA molecules are degraded and the peptide NLS·SZ will be degraded as well.

To test the influence of the destabilizing sequence on the output of the model, we have selected two variants, the first one where the NLS·SZ is stable, and the second one where the NLS·SZ is actively degraded, which increases the rate of this reaction by 100 fold (Supplementary Figure 5c). To estimate the stability of the UbiY-NLS-SZ in cells, we have performed an experiment where protein transcription is blocked by cycloheximide and the degradation kinetics of the protein can thus be quantified. A strain bearing the p*GPD1*-$dPSTR^R$ and p*GPD1*-$dPSTR^Y$ was grown overnight in SD-full containing 1M Sorbitol, diluted in the morning with the same medium, placed under the microscope and treated with 0.1mg/ml cycloheximide (Supplementary Figure 5d). In this high osmolarity medium, the basal expression level of p*GPD1* is increased due to the higher basal activity of Hog1. We measured a half-life of 2.1 ±0.5 min for the UbiY-NLS-SZ peptide which was used in the model. As expected and in agreement with our experimental measurements of the dPSTR, due to accumulation of all NLS·SZ expressed in the cell, the stable inducible peptide can lead to more saturation than the degraded one (Supplementary Figure 5c). However, the degraded peptide will be expressed at lower levels and thus nuclear enrichment of the fluorescent protein might be more difficult to detect at low concentrations. Therefore, the level of expression of the RFP·SZ might have to be adapted to accommodate the transcriptional output of the inducible promoter. We have achieved this by testing different combinations of ribosomal protein gene

promoters, which span a large range of expression levels[10] and different terminators[11]. Alternatively, for promoters with very low expression outputs, we could envision to multiply the SZ motif on the inducible protein, such that each expressed peptide would induce the recruitment of two or four fluorescent proteins.

Supplementary References

1. Pelet, S. *et al.* Transient activation of the HOG MAPK pathway regulates bimodal gene expression. *Science* **332,** 732–735 (2011).
2. Reinke, A. W., Grant, R. A. & Keating, A. E. A Synthetic Coiled-Coil Interactome Provides Heterospecific Modules for Molecular Engineering. *J. Am. Chem. Soc.* **132,** 6025–6031 (2010).
3. Thompson, K. E., Bashor, C. J., Lim, W. A. & Keating, A. E. SYNZIP Protein Interaction Toolbox: in Vitro and in Vivo Specifications of Heterospecific Coiled-Coil Interaction Domains. *ACS Synth. Biol.* **1,** 118–129 (2012).
4. Timney, B. L. *et al.* Simple kinetic relationships and nonspecific competition govern nuclear import rates in vivo. *J Cell Biol* **175,** 579–593 (2006).
5. Ghaemmaghami, S. *et al.* Global analysis of protein expression in yeast. *Nature* **425,** 737–741 (2003).
6. Wu, J.-Q. & Pollard, T. D. Counting cytokinesis proteins globally and locally in fission yeast. *Science* **310,** 310–314 (2005).
7. Zechner, C., Unger, M., Pelet, S., Peter, M. & Koeppl, H. Scalable inference of heterogeneous reaction kinetics from pooled single-cell recordings. *Nat Methods* **11,** 197–202 (2014).
8. Tyson, C. B., Lord, P. G. & Wheals, A. E. Dependency of size of Saccharomyces cerevisiae cells on growth rate. *J. Bacteriol.* **138,** 92–98 (1979).
9. Jorgensen, P., Nishikawa, J. L., Breitkreutz, B.-J. & Tyers, M. Systematic identification of pathways that couple cell growth and division in yeast. *Science* **297,** 395–400 (2002).
10. Knight, B. *et al.* Two distinct promoter architectures centered on dynamic nucleosomes control ribosomal protein gene transcription. *Genes & Development* **28,** 1695–1709 (2014).
11. Yamanishi, M. *et al.* A Genome-Wide Activity Assessment of Terminator Regions in Saccharomyces cerevisiaeProvides a "Terminatome" Toolbox. *ACS Synth. Biol.* **2,** 337–347 (2013).
